# Solar ultraviolet radiation exposure, and incidence of childhood (0–19 years) malignant and non-malignant brain tumour in a US population-based dataset, 2000–2021

**DOI:** 10.1007/s10654-025-01314-w

**Published:** 2025-11-24

**Authors:** Mark P. Little, Jim Z. Mai, Jill S. Barnholtz-Sloan, Martha S. Linet, Michelle Fang, Pavel Chernyavskiy, Victoria Kennerley, Elizabeth K. Cahoon, Myles G. Cockburn, Gerald M. Kendall, Michael G. Kimlin

**Affiliations:** 1 Radiation Epidemiology Branch, Division of Cancer Epidemiology and Genetics, National Cancer Institute, NIH, DHHS, Bethesda, MD 20892-9778 USA; 2 Faculty of Health, Life Sciences and Technology, Oxford Brookes University, Headington Campus, Oxford, OX3 0BP UK; 3 Feinberg School of Medicine, Northwestern University, Evanston, IL 60611 USA; 4 Center for Biomedical Informatics and Information Technology, Division of Cancer Epidemiology and Genetics, National Cancer Institute, NIH, DHHS, Bethesda, MD 20892-9778 USA; 5 Department of Public Health Sciences, University of Virginia School of Medicine, Charlottesville, VA 22908-0717 USA; 6 Biostatistics Branch, Division of Cancer Epidemiology and Genetics, National Cancer Institute, NIH, DHHS, Bethesda, MD 20892-9778 USA; 7 Department of Preventive Medicine, Keck School of Medicine, University of Southern California, 1975 Zonal Ave, Los Angeles, CA 90033 USA; 8 Cancer Epidemiology Unit, University of Oxford, Richard Doll Building, Old Road Campus, Headington, Oxford, OX3 7LF UK; 9 Faculty of Health and Medicine, Bond University, Robina, QLD 4229 Australia

**Keywords:** Non-ionising radiation, Ultraviolet radiation, Solar radiation, brain tumour, Childhood

## Abstract

Brain tumour is the second most common type of childhood cancer and the most common solid tumour in children, but its aetiology is largely unknown. Some previous studies have suggested that elevated ultraviolet radiation (UVR) exposures decrease brain tumour risk, but the evidence is inconsistent. We conducted a cross-sectional study (with census-derived population counts) to assess age < 20 malignant/non-malignant brain tumour incidence overall and for major categories in Surveillance, Epidemiology and End Results 2000–2021 data, using ground-based UVR-irradiance measures, via quasi-likelihood models accounting for over/under-dispersion, adjusted for age, sex, race/ethnicity and other socioeconomic variables. There were 29,088/18,585 cases of malignant/non-malignant brain tumour, with generally significant decreasing trends of both types of tumour with UVR irradiance [relative risk (RR) = 0.754/mW/cm^2^ (95% CI 0.659, 0.862, *p* < 0.0001) for malignant brain tumour, RR = 0.466/mW/cm^2^ (95% CI 0.382, 0.567, *p* < 0.0001] for non-malignant brain tumour), although there was significant heterogeneity by histopathologic subtype, race/ethnicity, and sex. Equally, there is a highly significant decreasing trend of both types of tumour with UVR-cumulative radiant exposure (*p* < 0.0001). These trends are also significant in many malignant/non-malignant brain tumour histopathological subtypes and racial/ethnic groups. However, there are certain non-malignant brain tumour subtypes, for example tumours of the pineal region and meningeal tumours, where RR significantly exceed 1 in relation to UVR irradiance (*p* = 0.0330, *p* = 0.0024 respectively). Our finding, of a generally protective effect of UVR on brain tumour risk is not clear-cut, and warrants large studies of specific histopathological pediatric/adolescent brain tumours using individual-level data on solar exposures and key effect modifiers and potential confounders.

**Supplementary Information:**

The online version contains supplementary material available at 10.1007/s10654-025-01314-w.

## Introduction

Brain tumour, whether malignant or non-malignant, is the second most common cancer in childhood in developed countries and the most common type of solid tumour in children [1, 2]. There has been a lack of improvement in survival and mortality over many years for childhood brain tumour and substantial morbidity of those who do survive [3, 4]. Brain tumour rates in childhood and young adulthood are generally stable in many countries [5, 6]. Despite decades of epidemiological observational studies, few consistently validated risk factors have been identified for childhood brain tumours (CBT) [7]. A 2024 systematic review and meta-analysis of 85 case–control and 96 cohort studies (1976–2022) revealed several associations based on varying numbers of studies, although there was some heterogeneity in results between and within study designs [8]. Onyije et al. [8] reported that childhood exposure to moderate-to-high-dose radiation and CT scans have been consistently associated with increased risk of CBT in cohort studies, and data from cohort, case–control and combined studies have also observed elevated risks linked with low (< 2500 g) and high birthweight (> 4000 g). Meta-analysis of varying numbers of cohort studies revealed elevated risks for maternal smoking > 10 cigarettes/day during pregnancy, paternal occupational exposure to paint before conception, caesarean section birth, gestational age > 40 weeks, large for gestational age at birth, large head circumference (> 38 cm) and use of artificial reproductive technology [8]. From case–control studies meta-analysis found associations with maternal residential exposure to insecticides during pregnancy, maternal consumption of cured, meat, and ≥ 2 cups of coffee/day and paternal occupational exposure to general pesticides, benzene, and wood dust [8]. Most of the pregnancy, periconception and post-natal factors linked with CBT were based on interview data. About 4% of CBT are due to rare single gene disorders or syndromes including neurofibromatosis Types 1 and 2, tuberous sclerosis complex (*TSC1* or *TSC2*), Li-Fraumeni (*TP53*), Gorlin syndrome (*PTCH1*), and others [9]. There is limited knowledge on genetic variants that may contribute since generally small genetic association studies have been conducted on brain tumours in children and adolescents. Preliminary evidence suggests that the rare germline variants are mainly located on genes involving DNA repair and cell cycle pathways predominantly in the *TP53* and *NF1* genes[9]. There are also significant associations of brain tumour with sex and racial/ethnic group, with White non-Hispanics at consistently higher risk for most types of tumour [7, 9]. The only likely protective factors, although mostly based on risk of glioma in adulthood, are allergies and atopy [7, 9, 10]. There is urgent need for identification of new aetiologic clues for prevention given the notable mortality and morbidity associated with childhood and adolescent brain tumours.

A meta-analysis of 75 cancer registries suggested an increase of childhood brain and spinal neoplasm incidence with increasing latitude, consistent with a protective effect of solar UVR exposure [11]. However, a case–control study of early childhood (ages 0–5) cancers nested within the California cancer registry, and using ground based assessments of UVR exposure, suggested a small increase in intracranial/intraspinal embryonal tumours (*n* = 550 cases) among those with highest level of UVR exposure, although there was no relation with UVR exposure for other intracranial and intraspinal tumours [12]. An ecological Spanish study found that all-age brain cancer mortality was inversely correlated with mortality from non-melanoma skin cancer [13], implying (since non-melanoma skin cancer is consistently associated with long-term UVR exposure) that brain cancer mortality may be inversely related to UVR exposure. It is biologically not implausible that UVR could be protective, via augmentation of production of vitamin D [14].

In the current study we specifically address the hypothesis that UVR exposure may be protective for brain tumour in childhood. We analyse childhood brain tumour risk by histological subtype and grade in relation to ambient solar UVR exposure in the most current Surveillance, Epidemiology and End Results (SEER) data [2] aggregated at the county level. We link the SEER data to a database of ground-based solar exposure measurements. The methodology for deriving UVR exposure is as previously described [15–17]. This method has been used to analyse SEER childhood acute lymphocytic leukaemia and non-Hodgkin lymphoma data in relation to solar UVR exposure [18]. We shall consider two metrics of ambient solar exposure and assess associations of solar-associated risk of two major categories pediatric/adolescent brain tumours by histopathologic subtype, age at diagnosis, sex, and race/ethnicity.

## Materials and methods

### Study population

County level SEER22 data for cases diagnosed in 2000–2021 was used [2] in population-based SEER cancer registries, restricting to malignant and non-malignant brain tumour cases under the age of 20 (not inclusive), as was done in our previous study [18]. [Although there is no firm consensus the age of 20 is commonly assumed as the upper limit of childhood—for example the National Institutes of Health (NIH) often assumes a limit of 18 years, while the Food and Drug Administration (FDA) assumes a limit of 21 years.] We included registries pertaining to parts of the states of California, Connecticut, Georgia, Idaho, Illinois, Iowa, Kentucky, Louisiana, Massachusetts, New Jersey, New Mexico, New York, Texas, Utah and Washington; for reasons detailed in the Supplement A (Methods) certain other states in SEER22 (Alaska, Hawaii) were omitted. In the analytical cohort, there were a total of 1078 counties, with total population years (under age 20) ranging from 25 to 2.68 × 10^6^, with mean 3.67 × 10^4^. Brain tumour was defined by the neoplasm recode 2021, as set out in Supplement A Tables A1 and A2. These include the major histological groups defined by Price et al. [4] of (a) diffuse astrocytic and oligodendroglial tumours, (b) pilocytic astrocytoma, (c) other astrocytoma variants, (d) ependymal tumours, (e) other gliomas, (f) neuronal and mixed neuronal-glial tumours, (g) choroid plexus tumours, (h) tumours of the pineal region, (i) embryonal tumours, (j) tumours of cranial and paraspinal nerves, (k) tumours of the meninges (l) lymphomas, (m) other haematopoietic neoplasms (n) germ cell tumours, (o) tumours of the sellar region and (p) haemangioma and other unclassified tumours. The analysis was restricted to first primary neoplasms of the brain and central nervous system (CNS). These tumours include the categories of cranial and paraspinal nerve cell tumours of the CNS. Tumours of the peripheral and of the sympathetic nervous system (neuroblastoma) were not included because the latter have different patterns of occurrence, descriptive epidemiology and likely different aetiology than brain tumours of the CNS [19].The age, sex and major racial/ethnic groups used are set out in Supplement A.

As in the previous analysis, we restricted analysis to the four largest racial/ethnic groups available in the SEER data, namely non-Hispanic white, non-Hispanic black, Hispanic (all races), and non-Hispanic Asian or Pacific Islander persons. The remaining subtypes had only a comparatively small number of cases, 22 in all. Further details of groups excluded are detailed in the Supplement A (Methods).

#### Study design

The dataset is analysed cross-sectionally, with the disease rate (number of cases per population year count) within each calendar year analyzed via a generalized linear model; further statistical details are given below. The county population-year counts used in the calculation of population-years, that is the sum of the population in each year (broken down by covariates such as age, sex, etc.), somewhat analogous to person–years at risk, were based on the 2000 U.S. standard population (single ages to 84—Census P25-1130). Given the known difference in childhood cancer rates between these racial/ethnic groups [20], and the geographical heterogeneity of distribution of the various racial/ethnic groups, analysis of exposure response could be potentially confounded. We therefore adjusted for racial/ethnic group in all analyses. [21].

### Solar radiation exposure assessment

The AVerage daily total GLObal solar radiation (AVGLO) estimates that are employed are derived from the National Solar Radiation Database (NSRAD) produced by the National Renewable Energy Laboratory (NREL) under the US Department of Energy’s Resource Assessment Program. This is the largest ground-based solar measurement network in the US, containing statistical summaries computed from hourly measurement data (with some infilling for missing data) for 239 US radiation stations for the period 1961–1990 (the only years with data available to us), including monthly, yearly, and 30-year average global solar radiation measures, and gives estimates of ambient solar exposure cumulated over a day, measured in W hour/m^2^. We employ county-level interpolations developed by Tatalovich et al. [22] which deliver estimates of potential solar ambient irradiance (~ 100–3000 nm) at 1 km^2^ resolution in the mainland US. Linkage of SEER brain tumour incidence data to this interpolated AVGLO exposure database was via the county-level Federal Information Processing System (FIPS) code. Further details, in particular details of data mislinkage (because of incompleteness in either the SEER or AVGLO data), are given in the Supplement A (Methods).

Two candidate exposure metrics are suggested a priori, namely UVR irradiance (in units of mW/cm^2^), which is proportional to UVR power density on a surface, or UVR cumulative radiant exposure (in units of MJ/cm^2^), which is proportional to *cumulative* solar UVR energy deposition on a surface. These are measures of UVR exposure recommended by the Commission Internationale de l’Eclairage (CIE) [23]. As noted by Lea [24], it is instantaneous or cumulative UVR energy deposition on a surface that is likely to be of most biological relevance. As noted in the Supplement A (Methods) AVGLO is approximately proportional to the average total solar irradiance (in units of mW/cm^2^). We shall also use the measure of cumulative radiant exposure (in units of MJ/cm^2^). The derivation of both measures is explained in more detail in the Supplement A (Methods), and is as previously employed [15–17]. Specifically we employ Supplement A expression (A1) to assess UVR irradiance and Supplement A expression (A3) to assess cumulative UVR radiant exposure to each group of children residing in the geographic region covered by the US radiation station AVGLO estimate, not separately to individual children.

### Variable included in analysis

The list of county-level variables available to us and included in the analysis are listed in Supplement A Table A3. These include standard demographic variables such as age and sex, racial/ethnic group and calendar year, for which there is some evidence of association. We also include a number of county-level sociodemographic variables, which may be markers of dietary and other socioeconomically-associated factors possibly associated with brain tumour risk [7], such as median rent, mean income per capita, poverty rate, mean Supplemental Nutrition Assistance Program (SNAP) level, percentage diabetic, percentage obese, percentage urban, percentage white/black/Hispanic/Asian etc. which were derived from the County Health Rankings database [25]. These are all regarded as potential confounders. However, as noted below not all the latter sociodemographic variables were included in most analyses, because of correlations with measures of UVR. The selection of variables used for adjustment, subject to the restriction of lack of correlation with UVR, was via purely statistical means, as outlined below.

### Sensitivity analysis

Because of suggestions of variations in ascertainment over the last few years of follow-up, some of it possibly related to covid-19 [21], we performed sensitivity analysis with the last period of follow-up (2017–2021) removed. Because of doubts in as to the tissue of origin being outside the brain or central nervous system (CNS), we also conducted sensitivity analysis excluding lymphoma and other haemopoietic tumours (whose origin is haemopoietic), germ cell tumours (whose origin are the germ cells), haemangioma and other unclassified tumours (haemangiomas originate in the blood vessels). A less obvious target for exclusion are pituitary and other tumours of the sellar region, which originate in hormone producing cells and nerve fibers associated with the hypothalamus; additionally we exclude these tumours also. These tumour endpoints, with the additional restriction to those with > 300 cases are the basis of the trend risks by tumour endpoint presented in Table 4; in Fig. 2 we display exposure response plots for the principal histopathologic types given in Table 4 with the additional restriction to subtypes with > 1000 cases.

### Statistical analysis

We evaluated the relative risks of malignant and non-malignant brain and CNS tumours according to the major racial and ethnic groups due to the known differences in incidence of these groups [26] and the known clinical and biological differences in skin pigment diversity and its consequences on UVR impact [27]. Because of marked over- and under-dispersion, with variance in relation to the Poisson-expected rates multiplied by factors that are generally between 0.9 and 1.4 (i.e. 10% under-dispersion to 40% overdispersion) in certain race-sex subgroups for both disease endpoints, a quasi-likelihood model was used for all model fits and tests of significance [28]. The model assumes that the expected number of cases in the stratum with population-years $PY_{i}$, after UVR exposure, $H_{i}$ (using either irradiance or cumulative radiant exposure), with various other explanatory covariates, $X_{i} = (X_{ij} )$, is given by:

1 $$PY_{i} \exp [\alpha H_{i} + \sum\limits_{j} {X_{ij} \beta_{j} } ]$$

The population in each year and subgroup defined by the stratification is summed over each separate calendar year to give the population-year total $PY_{i}$ for that subgroup. Model fitting is performed in R [29] using the glm function. Other variables used for adjustment were taken from a set of demographic/socioeconomic variables measured at county level. The variables measured are described in Supplement A Table A3. To avoid variables that could potentially absorb (and thereby diminish) the effect of UVR exposure, we exclude any which had absolute value of the (Pearson) correlation with UVR irradiance of 0.1 or greater. In order to avoid over-parameterised models, the Akaike Information Criterion (AIC) [30, 31] was employed to select the optimal subset of descriptive variables from this set. A mixed forward–backward stepwise algorithm was used to select the set of variables minimising AIC, using R [29]. In order to test the effect of excluding those baseline variables with correlation > 0.1, this restriction was relaxed, and AIC used to select the optimal subset of descriptive variables again. We also performed sensitivity analysis via model fits in which the demographic/socioeconomic variables were omitted. Profile-likelihood confidence intervals (CI) were estimated from the quasi-likelihood [28]. To test for heterogeneity by malignant or non-malignant subtype restricted-maximum likelihood (REML) methods were employed by fitting to the ln[relative risks (RR)], using the R metafor package [32]. All statistical tests were two-sided.

### Code and data availability

All data and R code used for the analysis is available via online Supplement B.

### Patient and public involvement statement

The undertaking of this analysis was done in part because of the preliminary indications in other data that UVR exposure may be protective at least for paediatric brain tumours. The detrimental health effects of UVR are much better known and are of interest to the public. Once published, the findings will be shared on our LinkedIn feeds, which reach a wide array of audiences, and available once indexed on PubMed. Organizations like the National Cancer Institute rely on publications like ours to inform their patient-facing materials on health information websites such as cancer.gov.

## Results

In all that follows *p*-values are generally highly significant (*p* < 0.001). We specifically mention the few exceptions. Among the four largest racial/ethnic groups analysed here there are 29,275 malignant brain and CNS tumours and 18,671 non-malignant cases among a population with 882,780,127 population-years of follow-up; after eliminating counties with missing FIPS code, these totals were reduced to 29,088 malignant brain and CNS tumour cases and 18,585 non-malignant cases (reductions of 0.64% and 0.46% respectively) among a population with 875,379,635 population-years (a reduction of 0.83%) of follow-up (Tables 1–3).

**Table 1:** Demographic factors and absolute incidence risks of malignant and non-malignant brain and CNS tumour, in the SEER 22-registry data for the years 2000–2021

	Malignant			Non-malignant		
	Cases/population-years	Incidence rate/10^5^ person/year (95% CI)	Heterogeneity *p*-value	Cases/population-years	Incidence rate/10^5^ person/year (95% CI)	Heterogeneity *p*-value
			*Attained age*			
0–1	3547/84,208,311	4.212 (4.088, 4.339)	< 0.0001	1547/84,208,311	1.837 (1.741, 1.936)	
2–3	3801/84,521,083	4.497 (4.369, 4.628)		865/84,521,083	1.023 (0.952, 1.098)	
4–5	3457/85,172,850	4.059 (3.938, 4.182)		951/85,172,850	1.117 (1.043, 1.194)	
6–7	3229/85,282,068	3.786 (3.669, 3.905)		1092/85,282,068	1.280 (1.201, 1.363)	
8–9	2862/86,007,744	3.328 (3.219, 3.439)		1295/86,007,744	1.506 (1.420, 1.595)	< 0.0001
10–11	2701/88,858,151	3.040 (2.937, 3.144)		1415/88,858,151	1.592 (1.506, 1.682)	
12–13	2591/89,821,293	2.885 (2.785, 2.986)		1796/89,821,293	2.000 (1.903, 2.100)	
14–15	2537/89,444,142	2.836 (2.738, 2.937)		2647/89,444,142	2.959 (2.841, 3.081)	
16–17	2350/90,173,924	2.606 (2.512, 2.702)		3406/90,173,924	3.777 (3.644, 3.914)	
18–19	2013/91,890,069	2.191 (2.105, 2.278)		3571/91,890,069	3.886 (3.752, 4.024)	
			*Racial/ethnic group*			
White non-Hispanic	16,411/418,038,113	3.926 (3.870, 3.982)	< 0.0001	9632/418,038,113	2.304 (2.256, 2.353)	
Black non-Hispanic	3245/119,804,110	2.709 (2.622, 2.797)		2220/119,804,110	1.853 (1.773, 1.936)	
Hispanic (all races)	7740/276,300,069	2.801 (2.743, 2.860)		5729/276,300,069	2.073 (2.017, 2.131)	< 0.0001
Asian/Pacific Islander	1692/61,237,343	2.763 (2.641, 2.889)		1004/61,237,343	1.640 (1.535, 1.749)	
			*Sex*			
Female	13,254/427,337,044	3.102 (3.054, 3.150)	< 0.0001	10,608/427,337,044	2.482 (2.435, 2.530)	< 0.0001
Male	15,834/448,042,591	3.534 (3.484, 3.584)		7977/448,042,591	1.780 (1.742, 1.820)	
			*Median rent ($)*			
< 300	70/2,087,095	3.354 (2.693, 4.115)	0.0005	41/2,087,095	1.964 (1.420, 2.634)	
300–399	753/22,107,329	3.406 (3.191, 3.631)		467/22,107,329	2.112 (1.925, 2.311)	
400–499	1802/54,966,378	3.278 (3.143, 3.417)		1205/54,966,378	2.192 (2.070, 2.319)	
500–599	2895/83,586,974	3.463 (3.351, 3.579)		1849/83,586,974	2.212 (2.112, 2.315)	0.3542
600–699	3227/93,174,911	3.463 (3.357, 3.572)		2005/93,174,911	2.152 (2.058, 2.248)	
700–799	4589/135,458,470	3.388 (3.300, 3.477)		2815/135,458,470	2.078 (2.002, 2.156)	
≥ 800	15,752/483,998,478	3.255 (3.209, 3.301)		10,203/483,998,478	2.108 (2.067, 2.150)	
*Supplemental nutrition assistance program (SNAP)(monthly benefit, $)*						
< 100	3590/103,020,241	3.485 (3.382, 3.589)	< 0.0001	2361/103,020,241	2.292 (2.199, 2.387)	< 0.0001
100–199	10,639/326,905,787	3.254 (3.199, 3.311)		6547/326,905,787	2.003 (1.954, 2.052)	
200–299	12,048/358,371,889	3.362 (3.308, 3.417)		7682/358,371,889	2.144 (2.095, 2.193)	
300–399	1860/55,206,699	3.369 (3.232, 3.510)		1194/55,206,699	2.163 (2.041, 2.290)	
400–499	458/14,332,889	3.195 (2.937, 3.469)		287/14,332,889	2.002 (1.776, 2.247)	
500–599	299/10,725,721	2.788 (2.510, 3.085)		370/10,725,721	3.450 (3.105, 3.819)	
≥ 600	194/6,816,409	2.846 (2.497, 3.226)		144/6,816,409	2.113 (1.781, 2.483)	
*Calendar year*						
2000–2003	5157/156,423,460	3.297 (3.216, 3.379)	< 0.0001	0/156,423,460	0.000 (0.000, 0.000)	< 0.0001
2004–2006	5264/158,215,014	3.327 (3.246, 3.409)		3294/158,215,014	2.082 (2.017, 2.149)	
2007–2011	5557/160,491,787	3.462 (3.380, 3.546)		4003/160,491,787	2.494 (2.423, 2.567)	
2012–2016	6906/200,690,608	3.441 (3.368, 3.515)		5665/200,690,608	2.823 (2.755, 2.891)	
2017–2021	6204/199,558,766	3.109 (3.039, 3.180)		5623/199,558,766	2.818 (2.750, 2.887)	
*UVR cumulative radiant exposure *(MJ cm^−2^)						
< 0.05	3554/84,307,311	4.216 (4.092, 4.342)	< 0.0001	1549/84,307,311	1.837 (1.743, 1.935)	< 0.0001
0.05–0.09	4075/90,279,403	4.514 (4.390, 4.640)		944/90,279,403	1.046 (0.977, 1.117)	
0.10–0.14	4376/110,058,456	3.976 (3.871, 4.083)		1285/110,058,456	1.168 (1.102, 1.236)	
0.15–0.19	3165/86,207,534	3.671 (3.557, 3.788)		1300/86,207,534	1.508 (1.424, 1.596)	
0.20–0.24	2893/88,951,163	3.252 (3.147, 3.360)		1293/88,951,163	1.454 (1.372, 1.538)	
0.25–0.29	2818/93,020,087	3.029 (2.930, 3.131)		1834/93,020,087	1.972 (1.878, 2.068)	
≥ 0.30	8207/322,555,681	2.544 (2.495, 2.594)		10,380/322,555,681	3.218 (3.153, 3.283)	
			*UVR irradiance *(mW cm^*−2*^)			
< 0.60	1013/25,104,171	4.035 (3.814, 4.265)	< 0.0001	729/25,104,171	2.904 (2.695, 3.124)	
0.60–0.64	5360/154,472,937	3.470 (3.386, 3.555)		3406/154,472,937	2.205 (2.130, 2.281)	
0.65–0.69	5975/168,483,731	3.546 (3.465, 3.628)		4059/168,483,731	2.409 (2.335, 2.485)	
0.70–0.74	2399/70,344,240	3.410 (3.288, 3.536)		1351/70,344,240	1.921 (1.818, 2.027)	< 0.0001
0.75–0.79	4592/140,644,625	3.265 (3.180, 3.351)		2914/140,644,625	2.072 (1.996, 2.149)	
0.80–0.84	7119/226,645,003	3.141 (3.075, 3.208)		4180/226,645,003	1.844 (1.788, 1.902)	
0.85–0.89	1359/45,756,249	2.970 (2.829, 3.116)		1087/45,756,249	2.376 (2.235, 2.522)	
≥ 0.90	1271/43,928,679	2.893 (2.752, 3.040)		859/43,928,679	1.955 (1.825, 2.091)	

The univariate absolute risks are shown for a quasi-likelihood model. The 95% CI are quasi-likelihood-based. The heterogeneity *p*-values are derived from the likelihood ratio test

**Table 2:** Relative risks of malignant brain and CNS tumour in relation to solar ultraviolet irradiance. Unless otherwise stated all CI are profile-likelihood based

Subgroup	Cases/population-years	Fully-adjusted		Adjusted without demographic/socioeconomic variables	
		Relative risk/mW cm^−2^ (+95% CI)	*p*-value	Relative risk/mW cm^−2^ (+95% CI)	*p*-value
White non-Hispanic, male^a^	9022/214,572,499	0.803 (0.635, 1.033)	0.0865	0.818 (0.649, 1.029)	0.0863
White non-Hispanic, female^a^	7389/203,465,614	0.578 (0.434^b^, 0.770^b^)	0.0002	0.555 (0.429, 0.717)	< 0.0001
Black non-Hispanic, male^a^	1700/60,890,268	0.833 (0.446, 1.550)	0.5638	0.819 (0.449, 1.488)	0.5123
Black non-Hispanic, female^a^	1545/58,913,842	1.423 (0.747^b^, 2.709^b^)	0.2834	1.178 (0.653, 2.119)	0.5865
Hispanic, male^a^	4179/141,287,849	0.842 (0.602^b^, 1.179^b^)	0.3170	0.717 (0.529, 0.974)	0.0331
Hispanic, female^a^	3561/135,012,220	0.741 (0.538, 1.046)	0.0879	0.727 (0.527, 1.004)	0.0527
Asian/Pacific Islander, male^a^	933/31,291,975	1.086 (0.619, 1.903)	0.7744	0.922 (0.578, 1.474)	0.7349
Asian/Pacific Islander, female^a^	759/29,945,368	0.449 (0.185, 1.083)	0.0748	0.414 (0.195, 0.883)	0.0225
Total (white + black non-Hispanic, Hispanic, Asian/Pacific Islanders)^c^	29,088/875,379,635	0.754 (0.659^b^, 0.862^b^)	< 0.0001	0.714 (0.632, 0.807)	< 0.0001
		Alternative measure of irradiance used by Coste et al. [33]			
		Relative risk/100 J/cm^2^/day	*p*-value	Relative risk/100 J/cm^2^/day	*p*-value
		0.721 (0.617^b^, 0.842^b^)	< 0.0001	0.677 (0.588, 0.780)	< 0.0001

^a^Fully-adjusted models for age (10 group factor variable), percentage urban, percentage screened and Supplemental Nutrition Assistance Program (SNAP) in that order; models without demographic/socioeconomic adjustments adjust only for age

^b^Wald-based CI

^c^Fully-adjusted models for age (10 group factor variable), racial/ethnic group (4 group factor variable), sex, percentage urban, percentage screened, Supplemental Nutrition Assistance Program (SNAP) and age x sex in that order; models without demographic/socioeconomic adjustments adjust only for age, racial/ethnic group, sex, age x sex

**Table 3:** Relative risks of non-malignant brain and CNS tumour in relation to solar ultraviolet irradiance. Unless otherwise stated all CI are profile-likelihood based

Subgroup	Cases/Population-years	Fully-adjusted		Adjusted without demographic/socioeconomic variables	
		Relative risk/mW cm^−2^ (+ 95% CI)	*p*-value	Relative risk/mW cm^−2^ (+ 95% CI)	*p*-value
White non-Hispanic, male^a^	4298/214,572,499	0.523 (0.372, 0.742)	0.0004	0.509 (0.365, 0.708)	< 0.0001
White non-Hispanic, female^a^	5334/203,465,614	0.624 (0.433^b^, 0.900^b^)	0.0116	0.586 (0.418, 0.820)	0.0018
Black non-Hispanic, male^a^	920/60,890,268	0.578 (0.237, 1.399)	0.2248	0.570 (0.243, 1.333)	0.1954
Black non-Hispanic, female^a^	1300/58,913,842	0.602 (0.313, 1.150)	0.1248	0.442 (0.243, 0.802)	0.0072
Hispanic, male^a^	2310/141,287,849	0.496 (0.305, 0.840)	0.0092	0.404 (0.257, 0.636)	< 0.0001
Hispanic, female^a^	3419/135,012,220	0.340 (0.244, 0.474)	< 0.0001	0.289 (0.213, 0.392)	< 0.0001
Asian/Pacific Islander, male^a^	449/31,291,975	0.300 (0.115, 0.784)	0.0140	0.385 (0.163, 0.914)	0.0305
Asian/Pacific Islander, female^a^	555/29,945,368	0.080 (0.014, 0.451)	0.0041	0.090 (0.012, 0.662)	0.0181
Total (white + black non-Hispanic, Hispanic, Asian/Pacific Islanders)^c^	18,585/875,379,635	0.466 (0.382, 0.567)	< 0.0001	0.424 (0.348, 0.515)	< 0.0001
		Alternative measure of irradiance used by Coste et al.[33]			
		Relative risk/100 J/cm^2^/day	*p*-value	Relative risk/100 J/cm^2^/day	*p*-value
		0.413 (0.328, 0.519)	< 0.0001	0.370 (0.295, 0.464)	< 0.0001

^a^fully-adjusted models for age (10 group factor variable), calendar year, Supplemental Nutrition Assistance Program (SNAP), median rent, percentage Asian, percentage screened, percentage urban in that order; models without demographic/socioeconomic adjustments adjust only for age and calendar year

^b^Wald-based CI

^c^fully-adjusted models for age (10 group factor variable), calendar year, sex, racial/ethnic group (4 group factor variable), Supplemental Nutrition Assistance Program (SNAP), median rent, percentage Asian, percentage screened, percentage urban, age x sex, racial/ethnic group x sex, year x race in that order; models without demographic/socioeconomic adjustments adjust only for age, calendar year, sex, racial/ethnic group, age x sex, racial/ethnic group x sex, year x race

For malignant and non-malignant brain tumour there are highly significant effects of age (with risk for non-malignant brain tumour varying in a U-shaped manner, although for malignant brain tumour risk generally reduced with increasing age). There are also variations by sex, with malignant tumour risk for males ~ 1.14 × that for females, but non-malignant tumour risk for males ~ 0.72  × that for females, and by racial/ethnic group (Table 1). The patterns for median rent are more complex, with malignant and non-malignant brain tumour risk tending to be lower for higher median rent, and likewise for SNAP, where for malignant brain tumour risks tend to be lower for higher levels of benefit, but indications of a contrary trend for non-malignant brain tumour (Table 1). The heterogeneity *p*-values are generally highly significant, the exception being the variation of non-malignant brain tumour with respect to median rent (*p* = 0.3542) (Table 1). Supplement A Table A4 demonstrates that for most of the major histopathological tumour subtypes (all except malignant meningeal tumours) there are highly significant variations in incidence risk by age. Supplement A Table A5 suggests that this is also the case by racial/ethnic group, where for most histopathological subtypes (all except malignant meningeal tumours and other non-malignant astrocytoma variants) there are highly significant variations in incidence risk by race. The analysis of Supplement A Table A6 indicates that there is somewhat weaker evidence of differences between the sexes in incidence risk, about half (9/15) of the subtypes showing significant (*p* < 0.05) differences; for many endpoints risk for males exceeds that for females, although the opposite pattern is observed for other malignant gliomas, non-malignant meningeal tumours and non-malignant sellar region tumours.

The stepAIC algorithm suggests that the optimal background model for malignant brain tumour includes age, racial/ethnic group, sex, percentage urban, percentage screened, SNAP, and age x sex, in that order (Table 2); for non-malignant brain tumour the optimal set of explanatory variables are age, calendar year, sex, racial/ethnic group, SNAP, median rent, percentage Asian, percentage screened, percentage urban, age x sex, racial/ethnic group x sex and calendar year x racial/ethnic group, in that order (Table 3).

**Fig. 1:**
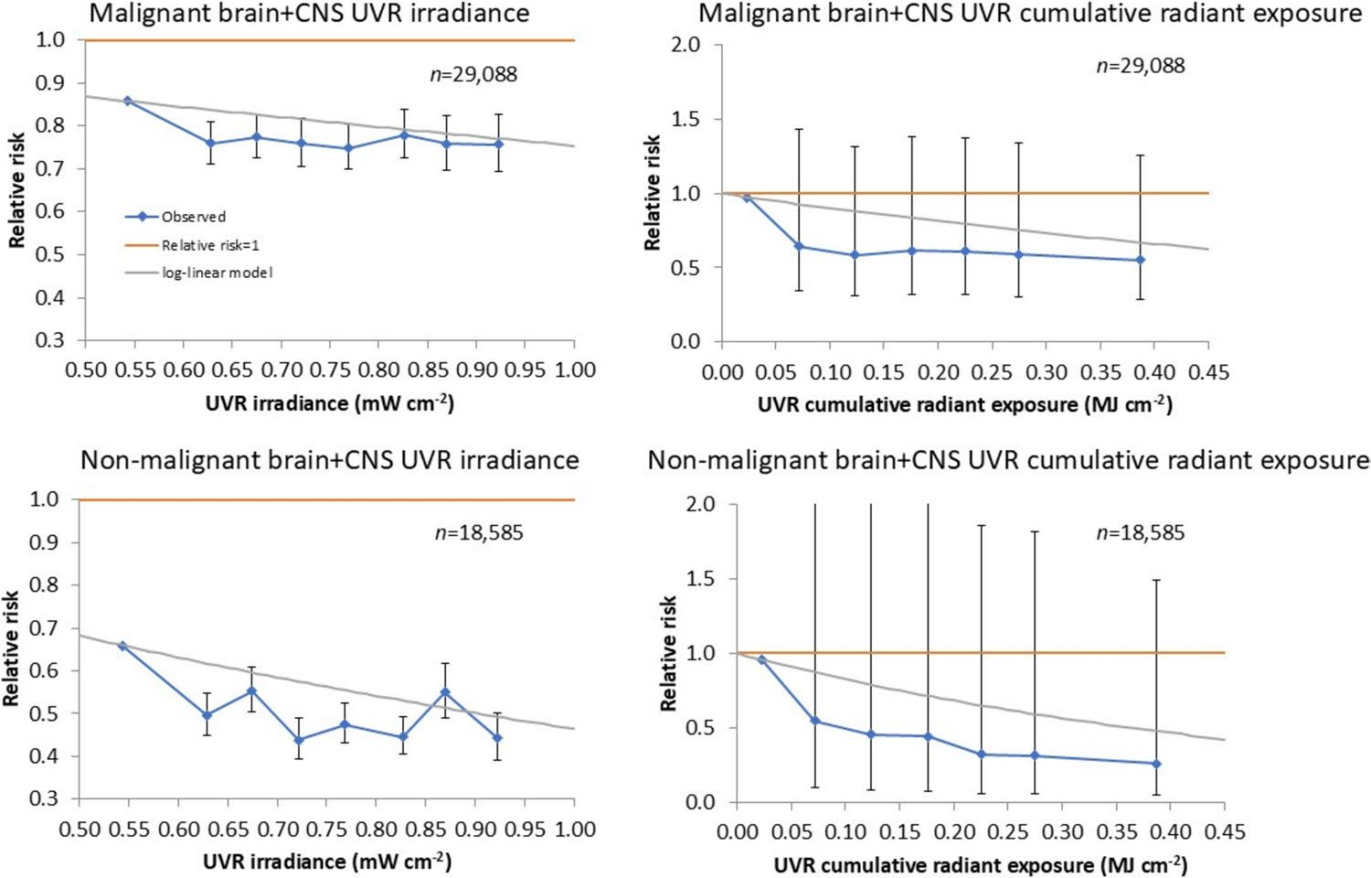
Relative risks (+ 95% CI) of **a** malignant brain and CNS tumour in relation to UVR irradiance, **b** malignant brain and CNS tumour in relation to UVR cumulative radiant exposure **c** non-malignant brain and CNS tumour in relation to UVR irradiance, **d** non-malignant brain and CNS tumour in relation to UVR cumulative radiant exposure. Fitted models are the most complete (all racial/ethnic group, both sexes) AIC-optimal models given in Tables 2 and 3

Table 2 and Fig. 1 demonstrate that using these models (and obvious simplifications [omitting the interaction terms in racial/ethnic group and sex] in the racial/ethnic group x sex subgroups) there is a highly significant inverse trend of malignant brain tumour with UVR irradiance, with relative risk (RR) = 0.754/mW/cm^2^ (95% CI 0.659, 0.862). There are decreasing trends of malignant brain tumour with UVR irradiance in white non-Hispanic females, and at borderline levels of significance for white non-Hispanic males (*p* = 0.0865) and Hispanic females (*p* = 0.0879) (Table 2). Equally, there is a highly significant decreasing trend of malignant brain tumour incidence with UVR cumulative radiant exposure, with RR = 0.357/MJ/cm^2^ (95% CI 0.242, 0.526), and again significant decreasing trends of brain tumour with UVR cumulative radiant exposure among white non-Hispanic females and Hispanic females (*p* = 0.0236), and at borderline levels of significance for white non-Hispanic males (*p* = 0.0813) (Supplement A Table A7). Very similar results are obtained if demographic/socioeconomic variables are not used for adjustment (Table 2, Supplement A Table A7).

Table 3 and Fig. 1 demonstrate that solar exposure also appears protective for non-malignant brain tumour, with decreases in incidence for increasing UVR irradiance with RR = 0.466/mW/cm^2^ (95% CI 0.382, 0.567). This is also observed in most racial/ethnic/sex subgroups, in particular in white non-Hispanic males and females (*p* < 0.001, *p* = 0.0116, respectively), in Hispanic males and females (*p* = 0.0092, *p* < 0.001, respectively) and in Asian and Pacific Islander males and females (*p* = 0.0140, *p* = 0.0041, respectively) (Table 3). There is also a highly significant decreasing trend of non-malignant brain tumour with UVR cumulative radiant exposure, with RR = 0.148/MJ/cm^2^ (95% CI 0.095, 0.232), with again highly significant decreasing trends in white non-Hispanic males and females, in Hispanic males and females (*p* = 0.0081, *p* < 0.001, respectively) and in Asian and Pacific Islander males and females (*p* = 0.0024, *p* = 0.0048, respectively) (Supplement A Table A8). Very similar results are obtained if demographic/socioeconomic variables are not used for adjustment (Table 3, Supplement A Table A8).

**Fig. 2:**
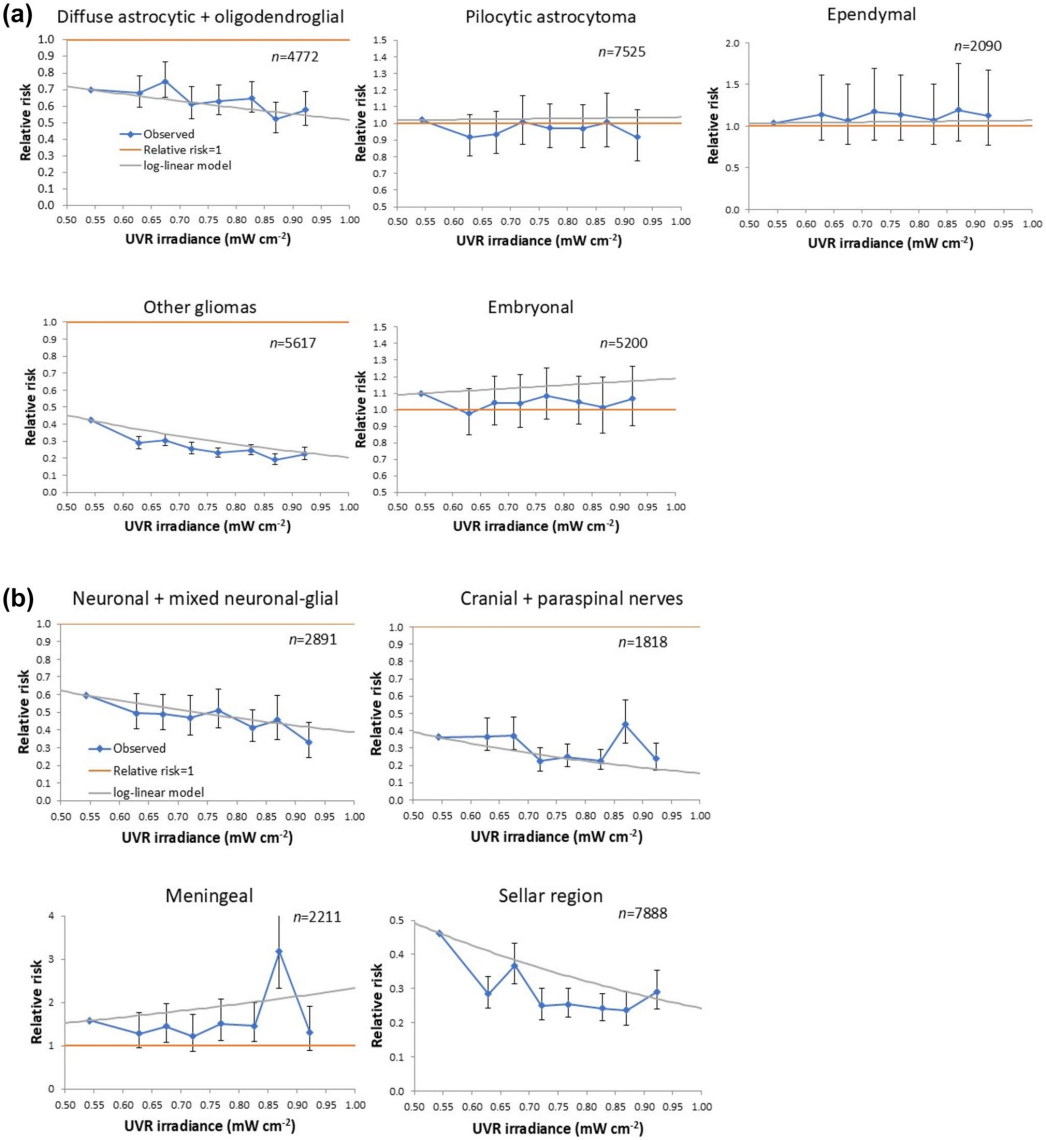
Relative risks (+ 95% CI) of the principal principal histopathologic subtypes (as in Table 4 with > 1000 cases) of **a** malignant and **b** non-malignant brain and CNS tumour in relation to UVR irradiance. The log-linear relative risk models plotted are as given in Tables 4, and the piecewise constant relative risk models are fitted adjusting for age [10-level factor], racial/ethnic group (4-level factor), calendar year and sex (as for the models in Table 4)

Table 4 and Supplement A Table A9 suggests that there is highly significant heterogeneity in UVR-related risk by malignant brain tumour subtype, although for many tumour subtypes [9 out of 17] RR are < 1, both in relation to UVR cumulative radiant exposure and UVR irradiance, even if not statistically significantly. However, as shown there and in Fig. 2 there are large groups of malignant tumours, for example pilocytic astrocytoma, ependymal tumour and embryonal tumour that do not exhibit a marked exposure response. Table 4 and Supplement A Table A9 also indicates that there is also highly significant heterogeneity in UVR-related risk by non-malignant brain tumour subtype, although again for many tumour subtypes (7/14) RR are < 1, both in relation to UVR cumulative radiant exposure and UVR irradiance. However, for certain non-malignant subtypes, for example pineal region tumours and meningeal tumours, RR significantly exceed 1, at least in relation to UVR irradiance (*p* = 0.0330, *p* = 0.0024 respectively), as also illustrated (for meningeal tumours) by Fig. 2. There is substantially greater improvement in fit using UVR irradiance compared with cumulative UVR radiant exposure for meningeal tumours, suggesting that irradiance may be the more relevant measure.

**Table 4:** Relative risks of principal histopathologic subtypes of malignant and non-malignant brain and CNS tumours cumulative UVR radiant exposure and UVR irradiance relative risk. We exclude showing the results for tumours with non-brain tissue origin or those with under 300 cases. These are given in Supplement A Table A9. Tumours are coded according to the classification given in Supplement A Tables A1 and A2. All relative risks are derived using a model adjusted for age (10-level factor), racial/ethnic group (4-level factor), calendar year and sex. All CI are derived from the profile likelihood. *p*-values for heterogeneity are derived via fitting a restricted-maximum likelihood model to the ln[RR] by endpoint

Tumour subtype	Cases	Cumulative radiant exposure		Irradiance		*p*-value heterogeneity [cumulative radiant exposure / irradiance]
		Relative risk/MJ cm^−2^ (+ 95% CI)	*p*-value	Relative risk/mW cm^−2^ (+ 95% CI)	*p*-value	
*Malignant brain tumours*						
Diffuse astrocytic and oligodendroglial tumours	4772	0.192 (0.097, 0.377)	< 0.0001	0.517 (0.396, 0.676)	< 0.0001	< 0.0001 / <0.0001
Pilocytic astrocytoma	7525	0.572 (0.265, 1.230)	0.1529	1.038 (0.804, 1.341)	0.7742	
Other astrocytoma variants	408	0.589 (0.110, 3.146)	0.5363	0.845 (0.407, 1.749)	0.6494	
Ependymal tumours	2090	0.583 (0.093, 3.630)	0.5635	1.070 (0.605, 1.891)	0.8168	
Other gliomas	5617	0.010 (0.004, 0.023)	< 0.0001	0.206 (0.157, 0.268)	< 0.0001	
Tumours of the pineal region	341	1.657 (0.081, 33.29)	0.7421	0.759 (0.264, 2.176)	0.6082	
Embryonal tumours	5200	1.544 (0.587, 4.056)	0.3781	1.189 (0.914, 1.547)	0.1964	
Tumours of meninges	333	0.373 (0.019, 7.269)	< 0.0001	0.726 (0.240, 2.193)	< 0.0001	
*Non-malignant brain tumours*						
Other astrocytoma variants	504	0.892 (0.027, 29.34)	0.9490	1.496 (0.474, 4.725)	0.4923	0.0005 / <0.0001
Ependymal tumours	363	0.031 (0.003, 0.363)	0.0057	0.171 (0.054, 0.540)	0.0026	
Neuronal and mixed neuronal-glial tumours	2891	0.109 (0.044, 0.270)	< 0.0001	0.387 (0.268, 0.558)	< 0.0001	
Choroid plexus tumours	534	1.786 (0.056, 56.27)	0.7422	0.667 (0.249, 1.786)	0.4208	
Tumours of cranial and paraspinal nerves	1818	0.022 (0.007, 0.073)	< 0.0001	0.156 (0.095, 0.254)	< 0.0001	
Tumours of meninges	2211	0.925 (0.247, 3.451)	0.9072	2.330 (1.349, 4.026)	0.0024	
Tumours of sellar region (including pituitary, craniopharyngioma)	7888	0.056 (0.027, 0.116)	< 0.0001	0.242 (0.169, 0.348)	< 0.0001

Supplement A Table A10 demonstrates that there is evidence of modification of trend RR by sex, for malignant brain tumour in relation to cumulative radiant exposure (both *p*-values < 0.05), although less strong indication of modification in relation to irradiance (*p* > 0.10). Likewise, there is highly significant modification of risk by race in relation to cumulative radiant exposure, but much less indication of modification in relation to irradiance (*p* > 0.2). By contrast Supplement A Table A10 demonstrates that there is little evidence of modification of trend RR by sex for non-malignant brain tumour (all *p-*values > 0.5), but strong indications of modification by race (all *p*-values < 0.05). The most pronounced inverse UVR irradiance trends RR for non-malignant brain tumour is for Asian and Pacific islanders with RR = 0.196/mW/cm^2^ (95% CI 0.089, 0.432), likewise the most pronounced UVR cumulative radiant exposure trend with RR = 0.076/MJ/cm^2^ (95% CI 0.040, 0.147) (Supplement A Table A11). For malignant brain tumour the inverse trends are more nearly equal for all ethnic groups apart from black non-Hispanics (Supplement A Table A11). The most marked inverse UVR irradiance trend RR for malignant brain tumour is for females with RR = 0.680/mW/cm^2^ (95% CI 0.564, 0.820), likewise the most pronounced UVR cumulative radiant exposure trend with RR = 0.224/MJ/cm^2^ (95% CI 0.127, 0.393) (Supplement A Table A12).

Supplement A Table A13 demonstrates that there is no significant variation in risk of malignant brain tumour risk in different age groups whether in relation to irradiance (*p* = 0.0984) or cumulative radiant exposure (*p* = 0.7885). Nevertheless, there is reduction (towards 1) in the inverse magnitude of the cumulative radiant exposure trend with increasing age, although there are indications of an opposite trend, with UVR-irradiance-related RR becoming increasingly protective with increasing age, in relation to irradiant UVR exposure. Supplement A Table A13 also shows that the evidence for such heterogeneity for non-malignant brain tumour is much stronger, at least for irradiance (*p* = 0.0075), although less so for cumulative radiant exposure (*p* = 0.3820). As for malignant brain tumour, it is notable that there are increasingly protective UVR-irradiance-related trends for non-malignant brain tumour with increasing age. Very similar results are obtained if the analyses omit adjustment for the various demographic/socioeconomic variables (Supplement A Table A13).

Supplement A Table A14 illustrates the effect of relaxing the restriction on baseline variables having correlation with UVR < 0.1. As can be seen, comparing also with the results in Tables 2 and 3, the effect of allowing these extra variables to be used is to generally weaken the inverse UVR-associated trends, particularly for malignant brain tumour in relation to irradiance or cumulative radiant exposure, and the trend for UVR irradiance is no longer significant (*p* = 0.1298). However, for non-malignant brain tumour less difference is made, and all trends remain highly significant. Supplement A Table A15 demonstrates that there is highly significant variation in risk of malignant brain tumour in different median rent groups in relation to irradiance (*p* = 0.001) although not in relation to cumulative radiant exposure (*p* = 0.1126). Supplement A Table A16 shows that the evidence for such heterogeneity for non-malignant brain tumour is non-significant, whether in relation to irradiance (*p* = 0.7917) or cumulative radiant exposure (*p* = 0.9672).

Supplement A Table A17 demonstrates that for malignant and non-malignant brain tumour omission of particular states has generally little effect on the regression with cumulative UVR radiant exposure. However, omission of California reduces the magnitude of the protective trend in relation to UVR irradiance, and to a lesser extent also for cumulative UVR radiant exposure, and statistical significance of the irradiance trend is lost (*p* = 0.9283 for malignant tumours, *p* = 0.1503 for benign tumours).

Supplement A Table A18 does not suggest any large effect of omitting the last few years of follow-up (2017–2021). Supplement A Table A19 does not suggest that exclusion of any of lymphoma and other haemopoietic tumours, germ cell tumours, haemangioma and other unclassified tumours, with or without additional exclusion for sellar region tumours have pronounced effect on trends with cumulative UVR radiant exposure or UVR irradiance, whether considering malignant or non-malignant tumours.

## Discussion

The present study has demonstrated a highly significant decrease in risk of both malignant and non-malignant brain and CNS tumour for age < 20 with increasing levels of ambient solar UVR (Table 2, Table 3, Supplement A Table A7, Table A8), although there is notable heterogeneity by histopathologic subtype, race/ethnicity, and sex (Table 4, Supplement A Tables A10, A11, A12). The inverse trends with UVR cumulative radiant exposure and UVR irradiance are seen for many specific subtypes of brain tumour, both malignant (9/17) and non-malignant (7/14). However, for certain non-malignant subtypes, for example pineal region tumours and meningeal tumours, RR significantly exceeds 1, at least in relation to UVR irradiance (Table 4, Supplement A Table A9, Fig. 2). The significance of the decreasing trend for malignant brain tumour is largely confined to the Hispanic and white non-Hispanic groups (Table 2, Supplement A Table A7), but for non-malignant brain tumour the inverse trend is apparent in all ethnic groups apart from Black non-Hispanic males (Table 3, Supplement A Table A8).

Our findings of racial/ethnic difference in brain tumour rates (Table 1) is supported by the systematic review of Nieblas-Bedolla et al. [26] of publications during 2005–2020 to determine racial and ethnic disparities in incidence, health care access and survival for children with CNS tumours in the US. They identified 30 studies, 10 of which focus on incidence differences and concluded that there were higher incidence of childhood brain tumours among White and Asian children than among Black, Hispanic and Native Americans. Linabery and Ross [34] found that overall incidence of CNS tumours over the period 1992–2004 in SEER data increased most among Hispanics.

Our findings of decreased risk of brain tumour with increasing solar exposure in childhood find some support from a meta-analysis of 75 cancer registries that suggested an increase of childhood brain and spinal neoplasm incidence with increasing latitude and with mean annual solar radiation levels, consistent with a protective effect of solar UVR exposure [11]. Set against these findings, a case–control study of early childhood (ages 0–5) tumours nested within the California cancer registry, and using, like the present study, AVGLO ground-based assessments of UVR exposure, suggested a borderline significant increase in intracranial/intraspinal embryonal tumours among those with the highest level (among four quartiles) of UVR exposure [12] (birth-year-adjusted OR for 4th quartile = 1.26, 95%CI 1.00–1.61; birth-year-, maternal age-, race- and child’s birth-year-adjusted OR for 4th quartile = 1.29, 95%CI = 1.01–1.65). In the same study there was no relation with UVR exposure for other intracranial and intraspinal tumours [12]. So this subgroup finding for embryonal tumours may have been due to chance. Because the analysis uses only quartiles of exposure it is also difficult to quantitatively compare their results with our own, a difficulty also with the other studies we discuss. It has also a much smaller number of cases, for example *n* = 550 embryonal tumours, compared with *n* = 5200 for this endpoint in our study (Table 4). Our findings are also to some extent consistent with a number of studies showing increases in all-age brain tumour risk with latitude [13, 35]. Other studies have examined brain tumour risk after various types of skin cancer, many of which are null [36, 37] although in one instance showing increased risk [38]. The relevance of these studies, all of brain cancer occurring after various types of skin cancer, and therefore in late adulthood, is questionable. A defect of all these studies is their ecological design. Apart from the Californian study [12] the measures of solar exposure are crude or non-existent, and other studies [13, 35, 38] are not confined to cancer in childhood. Latitude, which is used in some cases [13, 35], is only a proxy for UVR exposure, in particular will not take account of local climatic factors such as cloud cover or height above sea level, which are also known to be important. UVR is strongly associated with variations in latitude, and so it is possible that our findings may be driven by latitude, or some other factor that is strongly correlated with it.

A major strength, and relative novelty of our study is the use of two methods of measuring solar exposure. The use of these two UVR measures parallels a slightly earlier analysis of SEER acute lymphocytic leukaemia (ALL) and non-Hodgkin lymphoma (NHL) data [18], which has not otherwise been attempted hitherto. As set out by Douglas Lea [24] there are biophysical reasons why it is expected that these two measures of UVR energy deposition on a surface, that is cumulative energy deposited and rate of energy deposited, will be most relevant to biological endpoints. However, it is not clear which is most relevant here. Another major strength of our study is the large size, using prospectively gathered cancer status data, which is linked with an independent set of county-level solar exposure measures. The availability of a rich set of lifestyle and environmental measures, albeit ecological (measured at the level of county) is also a strength. Another major strength is our ability to examine the UVR effect in relation to different histological subtypes of brain tumour with varying aetiologies. The solar exposure measurements used in our study are based on interpolated solar exposure measurements derived from a 30-year series of measurements at 215 measurement stations distributed across the contiguous 48 US states [22].

What is used here is therefore a climatic average for a region and does not take account of year-to-year variations in solar exposure. These measurements cover the period 1961–1990 [22], so somewhat before the period of tumour incidence in our study, 2000–2021. However, there was no evidence of change over time in the solar measurement data [22, 39], so that it is likely that they will represent more current ambient solar exposure. As noted above our study is fundamentally ecological in design. It is well known that ecological studies can be prone to bias, because of variations of disease rate and exposure within areas, and the unavailability of individual (in particular within-area) adjustments for potentially confounding factors [40]. The spatial resolution, to the level of US counties is a potential limitation, although the evidence is that UVR does not vary much over even relatively large (100 km square) geographical units [41]. As such the potential for ecological bias may be limited. Solar exposure of an individual living at a specific location will exhibit much greater fluctuations than ambient variation because of differences in time spent outdoors and proximity to shade on different days throughout the year. Furthermore, the solar UVR exposure absorbed by the skin and vitamin D production, assuming that to be the relevant factor, will be further modified by the use of photoprotective agents such as hats, clothing and sunscreens in addition to epidermal melanin content. There is evidence that people tend to cover up more at lower latitudes [42], which implies that personal level exposures might be less than indicated by the ambient exposure data, suggesting a likely underestimation of the slope of the tumour-solar irradiance response. We also note that there is evidence for different use of sun protective measures amongst different racial/ethnic groups [43–46], with consistent evidence that Whites tend to exhibit higher rates of sun-protective measures than Hispanics and Blacks. This implies that the differences we found between rates in different racial/ethnic groups (Table 1) would be even more extreme were adjustment to be made for sun-protective measures, and would also imply that UVR-irradiance trends we have reported (Tables 2–4) would likely be biased towards the null. Our analyses (Supplement A Tables A15 and A16) demonstrate some evidence of socioeconomic modifiers of UVR irradiance risk at least for malignant brain tumour. The variation of solar exposure from year to year in this measurement set is relatively slight [22, 39] and climatic norms will give far more representative sunlight values for the region of interest. Given that disease counts and underlying estimates of populations in our database are available only for complete calendar years, this may not matter too much, but inevitably there will be inaccuracies in assessing exposure. The denominators used, derived from the 2000 US census, which were the latest available in the SEER data (see Supplement A), are possibly inaccurate, particularly in the later years of coverage.

As with all other studies of solar radiation our study does not take account of population migration. In our data, solar exposure is linked to the SEER county of residence at diagnosis. The effect of this is that a proportion of the population in each area, which would be larger with increasing age, will have expected solar exposure which in the worst case, of in-migration from anywhere within the US, will correspond to the US average, so that the variation in true solar exposure (of the underlying population) between areas will be to some extent over-estimated. There will also be Berkson errors resulting from applying the group means to the individual exposures, but at least to first order the effect of these on trend estimates will again be minimal [47], although uncertainties could be underestimated [48, 49]. That said, the great advantage of studying childhood cancers is that they occur early in life, so that the effects of population migration should not be too serious [50–52]. Indeed Bell and Belanger [51] reviewing the literature on residential mobility about the time of birth in various developed countries, noted explicitly that most mothers, when they move, stayed within the same county; there was little variation between countries in this respect. Another source of error is the determination of Hispanic origin in SEER. This employs an algorithm using the patient’s surname, so that misclassification of certain cases is possible. However, as such misclassification is unlikely to vary with degree of UVR exposure it will probably not introduce bias in assessments of cancer risk in relation to UVR exposure.

It is not known what the relevant exposure period for solar exposure is likely to be for the childhood cancers considered here. It is suspected that the relevant exposure for such cancers is very early in life [19, 53], although our analysis of interaction of UVR-associated risk with age does not suggest that this is the case (Supplement A Table A13). As such correlating with solar exposure at diagnosis, an inescapable feature of the SEER registry data is likely to be not altogether the correct thing to do. However, with childhood cancers one can be much more confident that the solar exposure being measured relates to the entire duration of life up to the point of development of cancer. There are some potential mechanisms for the findings that we observe. It is possible the protective effects of vitamin D are mediated via cellular gap-junctional mechanisms [54, 55], reinforced by findings of reductions in cancer mortality following vitamin D supplementation [56] although there no effects of supplementation on cancer incidence [57, 58]; however, the relevance of these trials is debatable, as all are of populations in adulthood, with no specific analysis of brain tumour. A case–control study of childhood brain tumours that evaluated neonatal blood spots taken at birth did not find any relation with circulating vitamin D, but the study is small (*n* = 247 cases), and probably statistically underpowered [59]. UVR, and specifically UVB is absorbed via 7-dehydrocholesterol (7DHC), which gets transformed into vitamin D3 [14]. However, UVB is known (from experimental data) to be active in absorption and transformation of a number of other chromophores, which modulate the action of the neuroendocrine, immune and cardiovascular systems [14], and the nervous system [60]. There may be other systemic effects of UVR exposure, which for erythemal exposures has a depressive effect on the immune system [61] although for sub-erythemal exposures UVR likely boosts the immune system [62–66]. Since atopy is one of the few known factors tending to reduce the risk of brain tumour [7, 9, 10] this may also imply another mechanism for a protective effect of at least sub-erythemal UVR exposure on brain tumour.

In summary, our findings of a protective effect of solar exposure on malignant and non-malignant brain tumour are generally supported by several previous, but generally lower-quality ecological studies. However, the evidence is not entirely clear-cut and all findings are in need of replication in large studies of specific histopathological pediatric/adolescent brain tumours using individual-level data on solar exposures (and associated behavioural factors and protective measures) that can be assigned more accurately and in which information on effect modifiers (sex, race/ethnicity) and known/suspected confounding factors is available.

## Supplementary Information

Below is the link to the electronic supplementary material.

Supplementary file1 (DOCX 155 KB)

Supplementary file2 (ZIP 67484 KB)

## Data Availability

All data and R code used for the analysis is available via online Supplement B.
