# Supplementary material for: Solar ultraviolet radiation exposure, and incidence of childhood (0–19 years) malignant and non-malignant brain tumour in a US population-based dataset, 2000–2021
Source: Eur J Epidemiol. 2025 Nov 24;41(3):351–66. doi: 10.1007/s10654-025-01314-w (PMC13222178; doi:10.1007/s10654-025-01314-w)
Supplement: Supplementary file 1 — Supplementary file1 (DOCX 155 KB) [file 10654_2025_1314_MOESM1_ESM.docx]

**Supplement A**

**Supplemental Methods and Tables.**

**Supplemental Methods**

*Study population*

County level SEER22 data for cases diagnosed in 2000-2021 was used (1) in population-based SEER cancer registries, restricting to ALL and NHL cases under the age of 20 (not inclusive). The SEER22 data included registries pertaining to parts of the states of California, Connecticut, Georgia, Idaho, Illinois, Iowa, Kentucky, Louisiana, Massachusetts, New Jersey, New Mexico, New York, Texas, Utah and Washington; as detailed below certain other states in SEER22 were omitted for various reasons. There are a total of 1078 counties in the analytical dataset after AVGLO linkage (as detailed below).

The codes used to define malignant and non-malignant brain tumour subtypes are set out in Table A1 and A2.

*Measures of UVR exposure*

*Solar radiation exposure assessment*

The AVerage daily total GLObal solar radiation (AVGLO) estimates that are employed are derived used the National Solar Radiation Database (NSRAD) produced by the National Renewable Energy Laboratory (NREL) under the US Department of Energy’s Resource Assessment Program. This is the largest ground-based solar measurement network in the US, containing statistical summaries computed from hourly measurement data (with some infilling for missing data) for 239 US radiation stations for the period 1961-1990, including monthly, yearly, and 30-year average global solar radiation measures. We employ interpolations developed by Tatalovich *et al* (2) which deliver estimates of potential solar ambient irradiance (~100-3000 nm) at 1 km² resolution in the mainland US. Linkage of SEER data to this interpolated AVGLO exposure database was via the county-level Federal Information Processing System (FIPS) code.

Using the standard Commission Internationale de l’Eclairage (CIE) (International Commission on Illumination) terminology (3) we estimate the UVR (=UVA+UVB) irradiance in mW/cm^2^ from the AVGLO measure $Z$ of solar daily radiant exposure in W hour/day/m^2^ estimated by Tatalovich *et al* (2) via the conversion factor outlined by Little *et al* (4), namely:

$W=\frac{Z}{24\cdot10F_{tot/UVR}}$ (A1)

where $F_{tot/UVR}$ is the ratio of total (ground-level) solar output to UVR, which we estimate as $F_{tot/UVR}\approx1/0.04=25$ (5). Coste *et al* (6) use a closely related measure, the daily average UVR radiant exposure in J/cm^2^/day for a specified location, which is given by:

$P=\frac{24\text{ x }3600\text{ x }Z}{10000\text{ x }24\text{ x }F_{tot/UVR}}=\frac{0.36Z}{F_{tot/UVR}}$ (A2)

It will also be of interest to estimate the cumulative radiant exposure (in MJ/cm^2^) up to a specified age $a$ (in years), with lag period $t_{lag}$ given by:

$E(a)=\frac{Z(3600\text{ x }24\text{ x }365.2425\text{ x max[}a-t_{lag},0])}{24\text{ x }F_{tot/UVR}\text{ x }10^{10}}=\frac{Z(3.6\text{ x }365.2425\text{ x max[}a-t_{lag},0])}{F_{tot/UVR}\text{ x }10^{7}}$ (A3)

Based on the given AVGLO values given this would suggest values of irradiance of 0.683 mW / cm^2^ for Washington DC, 0.651 mW / cm^2^ for Boston (Suffolk county) and 0.776 mW / cm^2^ for Houston, TX (Houston county), while cumulative UVR radiant exposure at age 10 would be 0.216 MJ / cm^2^ for Washington DC, 0.206 MJ / cm^2^ for Boston and 0.245 MJ / cm^2^ for Houston, TX. In Tables 1-4, Supplement A Tables A4-A19 and Figure 1, Figure 2 we give risks for malignant and non-malignant brain and CNS tumours in relation to these derived quantities.

*Data restrictions*

We restricted analysis to the four main racial/ethnic groups, namely white non-Hispanic, black non-Hispanic, Hispanic (all races), and non-Hispanic Asian or Pacific Islanders. The cross classification also used age (grouped as 0-1, 2-3, 4-5, 6-7, 8-9, 10-11, 12-13, 14-15, 16-17, 18-19 years), sex (male, female) and calendar-year of follow up (grouped as 2000-2003, 2004-2006, 2007-2011, 2012-2016, 2017-2021). The full data that would allow us to separate Asians and Pacific Islanders in the analysis is not available for anything save a subset (2000-2014) of the data. Nevertheless it is known that of the malignant brain tumour cases 92.1% are Asian and 7.9% Pacific Islander; no such information is available on the non-malignant tumour cases. We excluded Alaska’s registry because Alaska was a UVR outlier and only Alaskan Natives were included, in addition to excluding non–Hispanic American Indian/Alaska Native; there were 138 cases of malignant brain tumour, 149 cases of non-malignant brain tumour in a population-year total of 6,352,233. The Hawaii registry cases were excluded because there is no AVGLO exposure estimate for them; there were 150 malignant brain tumour cases and 85 non-malignant brain tumours, in a population-year total of 7,400,492. There were 13 counties for which AVGLO database linkage could not be made, in 11 cases because the county was labelled as “unknown” (although with known state) in the SEER data, and for two because the FIPS code did not exist in the AVGLO database. As in the previous analysis, we restricted analysis to the four largest racial/ethnic groups, namely non-Hispanic white, non-Hispanic black, Hispanic (all races), and non-Hispanic Asian or Pacific Islander persons. The remaining subtypes had only a comparatively small number of cases, 22 in all. In the analysis dataset we excluded cells with 0 population-years, as not so doing would have resulted in model fitting errors. The county population-year counts used in the calculation of population-years (somewhat analogous to person–years) at risk were based on the 2000 U.S. standard population (single ages to 84 – Census P25-1130). Given the known difference in childhood cancer rates between these racial/ethnic groups (7), and the geographical heterogeneity of distribution of the various racial/ethnic groups, analysis of exposure response could be potentially confounded. We therefore adjusted for racial/ethnic group in all analyses. Measures of ethnicity and racial group are SEER derived; in particular Hispanic ethnicity is derived from the surname. It was judged that there were insufficient non-white Hispanic cases to justify division of the Hispanic population into white and non-white. For example, of 6585 Hispanic malignant brain tumour cases, excluding unknown only 308 (4.8%) were non-white. The denominators used are the population totals by area, age, sex and racial/ethnic groups derived from the 2000 US census information, the latest data available on SEER.

Brain tumour was defined by the neoplasm recode 2021, as set out in Supplement A Tables A1 and A2. These include the major histological groups of (a) diffuse astrocytic and oligodendroglial tumours, (b) pilocytic astrocytoma, (c) other astrocytoma variants, (d) ependymal tumours, (e) other gliomas, (f) neuronal and mixed neuronal-glial tumours, (g) choroid plexus tumours, (h) tumours of the pineal region, (i) embryonal tumours, (j) tumours of cranial and paraspinal nerves, (k) tumours of the meninges (l) lymphomas, (m) other haematopoietic neoplasms (n) germ cell tumours, (o) tumours of the sellar region and (p) haemangioma and other unclassified tumours.

*Statistical Analysis*

Because of marked departures from Poisson dispersion (with both under- and over-dispersion), a quasi-likelihood model was used for all model fits and tests of significance (8). The model assumes that the expected number of cases in the stratum with population-years $PY_{i}$, after UVR exposure, $H_{i}$ (using either irradiance or cumulative radiant exposure), with various other explanatory covariates, $X_{i}=(X_{ij})$, is given by:

$PY_{i}\exp[\alpha H_{i}+\sum_{j} X_{ij}\beta_{j}]$ (A4)

The population in each year and subgroup defined by the stratification is summed over each separate calendar year to give the population-year total $PY_{i}$ for that subgroup. As described above, we also employ the units of daily radiant exposure of J/cm^2^/day used by Coste *et al* (6). Model fitting is performed in R (9) using the glm function. Other variables used for adjustment were taken from a set of demographic/socioeconomic variables measured at county level derived from the County Health Rankings database (10). The variables measured are described in Table A1, a mixture of socioeconomic variables (median rent, Supplemental Nutrition Assistance Program (SNAP)) and other areal descriptors (e.g., percentage Hispanic, percentage urban, percentage colon cancer screened). Percentage colon cancer screened is a measure of the diagnostic medical facilities available in an area, and also of the population’s awareness of such screening tools, thereby representing a measure of deprivation. To avoid variables that could potentially soak up the effect of UVR exposure, we exclude any which had absolute value of the (Pearson) correlation with UVR irradiance of 0.1 or greater. In order to avoid over-parameterised models, the Akaike Information Criterion (AIC) (11, 12) was employed to select the optimal subset of descriptive variables from this set. A mixed forward-backward stepwise algorithm was used to select the set of variables minimising AIC, using R (9). In order to test the effect of excluding those baseline variables with correlation >0.1, this restriction was relaxed, and AIC used to select the optimal subset of descriptive variables again. We also performed sensitivity analysis via model fits in which the demographic/socioeconomic variables were omitted. Profile-likelihood confidence intervals (CI) were estimated from the quasi-likelihood (8). All statistical tests were two-sided. All R code used is given in Supplement B.

**Table A1 Brain and Other Central Nervous System Tumour Topography Groupings (based on Price *et al* (13)), 2000-2021 SEER 22**

| Site | ICD-O-3 Site code^a^ |
| --- | --- |
| Nasal cavity^b^ | C30.0 |
| Meninges (cerebral & spinal) | C70.0-C70.9 |
| *Cerebral meninges* | C70.0 |
| *Spinal meninges* | C70.1 |
| *Meninges, NOS* | C70.9 |
| Cerebrum | C71.0 |
| Frontal lobe of brain | C71.1 |
| Temporal lobe of brain | C71.2 |
| Parietal lobe of brain | C71.3 |
| Occipital lobe of brain | C71.4 |
| Ventricle | C71.5 |
| Cerebellum | C71.6 |
| Brainstem | C71.7 |
| Other brain | C71.8-C71.9 |
| *Overlapping lesion of brain* | C71.8 |
| *Brain, NOS* | C71.9 |
| Spinal cord and cauda equina | C72.0-C72.1 |
| *Spinal cord* | C72.0 |
| *Cauda equina* | C72.1 |
| Cranial nerves | C72.2-C72.5 |
| *Olfactory nerve* | C72.2 |
| *Optic nerve* | C72.3 |
| *Acoustic nerve* | C72.4 |
| *Cranial nerve, NOS* | C72.5 |
| Other nervous system | C72.8-C72.9 |
| *Overlapping lesion of brain and central*  *nervous system* | C72.8 |
| *Nervous system, NOS* | C72.9 |
| Pituitary and craniopharyngeal duct | C75.1-C75.2 |
| *Pituitary gland* | C75.1 |
| *Craniopharyngeal duct* | C75.2 |
| Pineal gland | C75.3 |

^a^International Classification of Diseases for Oncology, 3rd Edition, 2000 . World Health Organization, Geneva, Switzerland (14).

^b^ICD-O-3 histopathology codes 9522-9523 only.

**Table A2 Brain and Other Central Nervous System Tumour Histopathology Groupings (based on Price *et al* (13)), 2000-2021 SEER 22**

| **Histopathology** | **ICD-O-3**[^a^](javascript:;)**Histopathology and Behaviour Code**[^b^](javascript:;) | |
| --- | --- | --- |
|  | **Malignant (2000-2021)** | **Non-Malignant (2004-2021)** |
| Diffuse Astrocytic and Oligodendroglial Tumours | 9381/3, 9400/3, 9401/3, 9410/3, 9411/3, 9420/3, 9440/3, 9441/3, 9442/3, 9445/3, 9450/3, 9451/3, 9460/3, 9382/3 | 9442/1 |
| Pilocytic astrocytoma* | 9421/1^c^, 9421/3, 9425/3 | *None* |
| Other astrocytoma variants* | 9424/3 | 9384/1, 9431/1 |
| Ependymal Tumours* | 9391/3, 9392/3, 9393/3, 9396/3 | 9383/1, 9391/1 (excluding site C75.1), 9394/1 |
| Other Gliomas | 9380/3, 9385/3, 9423/3, 9430/3 | 9444/1 |
| Neuronal and Mixed Neuronal-Glial Tumours* | 8680/3, 8693/3, 9490/3, 9505/3, 9509/3, 9522/3 (site C30.0 only), 9523/3 (site C30.0 only) | 8680/0,1, 8681/1, 8690/1, 8693/1, 9412/1, 9413/0, 9490/0, 9492/0 (excluding site C75.1), 9493/0, 9505/0,1, 9506/1, 9509/1 |
| Choroid Plexus Tumours | 9390/3 | 9390/0,1 |
| Tumours of The Pineal Region | 9362/3, 9395/3 | 9360/1, 9361/1 |
| Embryonal Tumours | 8963/3, 9364/3, 9470/3, 9471/3, 9472/3, 9473/3, 9474/3, 9475/3, 9476/3, 9477/3, 9478/3, 9480/3, 9500/3, 9501/3, 9502/3, 9508/3 | *None* |
| Tumours of Cranial and Paraspinal Nerves | 9540/3, 9560/3, 9561/3, 9571/3 | 9540/0,1, 9541/0, 9550/0, 9560/0,1, 9562/0, 9563/0, 9570/0, 9571/0 |
| Tumours of Meninges | 8710/3, 8711/3, 8720/3, 8728/3, 8770/3, 8800/3, 8801/3, 8802/3, 8803/3, 8804/3, 8805/3, 8806/3, 8810/3, 8811/3, 8815/3[^c^](javascript:;), 8825/3, 8830/3, 8840/3, 8850/3, 8851/3, 8852/3, 8853/3, 8854/3, 8857/3, 8890/3, 8900/3, 8901/3, 8902/3, 8910/3, 8912/3, 8920/3, 8921/3, 8935/3, 8990/3, 9040/3, 9120/3, 9130/3, 9150/3, 9170/3, 9180/3, 9220/3, 9231/3, 9240/3, 9243/3, 9260/3, 9370/3, 9371/3, 9372/3, 9530/3, 9537/3, 9538/3, 9539/3 | 8711/0, 8728/0,1, 8800/0, 8810/0, 8811/0, 8815/0,1[^c^](javascript:;), 8821/1, 8824/0,1, 8825/0,1, 8830/0,1, 8831/0, 8835/1, 8836/1, 8840/0, 8850/0,1, 8851/0, 8852/0, 8854/0, 8861/0, 8870/0, 8890/0, 8897/1, 8900/0, 8920/1, 8935/0,1, 8990/0,1, 9040/0, 9120/0, 9125/0, 9130/0,1, 9131/0, 9136/1, 9150/0,1, 9161/0,1, 9170/0, 9180/0, 9210/0, 9220/0, 9241/0, 9530/0,1, 9531/0, 9532/0, 9533/0, 9534/0, 9535/0, 9537/0, 9538/1, 9539/1 |
| Lymphoma | 9590/3, 9591/3, 9596/3, 9650/3, 9651/3, 9652/3, 9653/3, 9654/3, 9655/3, 9659/3, 9661/3, 9662/3, 9663/3, 9664/3, 9665/3, 9667/3, 9670/3, 9671/3, 9673/3, 9675/3, 9680/3, 9684/3, 9687/3, 9688/3, 9690/3, 9691/3, 9695/3, 9698/3, 9699/3, 9701/3, 9702/3, 9705/3, 9712/3, 9714/3, 9715/3, 9719/3, 9724/3, 9727/3, 9728/3, 9729/3, 9735/3, 9737/3, 9738/3, 9750/3, 9751/3, 9755/3, 9756/3, 9811/3, 9812/3, 9813/3, 9814/3, 9815/3, 9816/3, 9817/3, 9818/3, 9819/3, 9823/3, 9826/3, 9827/3, 9831/3, 9832/3, 9837/3, 9861/3, 9866/3, 9930/3, 9965/3, 9966/3, 9967/3, 9971/3, 9975/3 | 9751/1, 9970/1 |
| Other haematopoietic neoplasms | 9731/3, 9733/3, 9734/3, 9740/3, 9741/3, 9749/3, 9753/3, 9754/3, 9757/3, 9758/3, 9759/3, 9760/3, 9766/3, 9860/3 | 9752/1, 9766/1 |
| Germ Cell Tumours | 8440/3, 9060/3, 9061/3, 9064/3, 9065/3, 9070/3, 9071/3, 9072/3, 9080/3, 9081/3, 9082/3, 9083/3, 9084/3, 9085/3, 9100/3, 9101/3 | 8440/0, 9080/0,1 |
| Tumours of Sellar Region (including pituitary, craniopharyngioma) | 8140/3, 8246/3, 8260/3, 8270/3, 8272/3, 8280/3, 8281/3, 8290/3, 8300/3, 8310/3, 8323/3, 9580/3 | 8040/0,1, 8140/0,1, 8146/0, 8260/0, 8270/0, 8271/0, 8272/0, 8280/0, 8281/0, 8290/0, 8300/0, 8323/0, 9350/1, 9351/1, 9352/1, 9391/1 (site C75.1 only), 9432/1, 9492/0 (site C75.1 only), 9580/0, 9582/0 |
| Hemangioma, other and unclassified tumours | 8000/3, 8001/3, 8002/3, 8003/3, 8004/3, 8005/3, 8010/3, 8020/3, 8021/3, 8320/3, 8452/3, 8896/3, 8980/3, 9133/3, 9140/3, 9503/3 | 8000/0,1, 8001/0,1, 8005/0, 8010/0, 8713/0, 9084/0, 9121/0, 9122/0, 9123/0, 9133/1, 9173/0, 9363/0 |

^a^International Classification of Diseases for Oncology, 3rd Edition, 2000. World Health Organization, Geneva, Switzerland(14).

^b^See the CBTRUS website for additional information about the specific histopathology codes included in each group: [http://www.cbtrus.org](http://www.cbtrus.org/).

^c^Added starting with diagnosis year 2018.

* All or some of this histopathology is included in the CBTRUS definition of gliomas, including ICD-O-3 histopathology codes 9380-9384, 9391-9460.

Abbreviations: WHO, World Health Organization; NOS, not otherwise specified.

**Table A3. Variables potentially used to adjust baseline risk for malignant and non-malignant brain and CNS tumour**

| **Variable** | **Notes** |
| --- | --- |
| **Standard demographic variables and their interactions** | |
| Age | factor variable with 10 groups |
| Sex | factor variable with 2 groups (male, female) |
| Racial/ethnic group | factor variable with 4 groups (white non-Hispanic, black non-Hispanic, Hispanic, Asian and Pacific islanders) |
| calendar year | factor variable with 7 groups – but analyzed as continuous variable using midpoint of each interval |
| age x sex |  |
| age x racial/ethnic group |  |
| age x calendar year |  |
| sex x racial/ethnic group |  |
| sex x calendar year |  |
| Racial/ethnic group x calendar year |  |
| **Sociodemographic variables with abs(correlation with UVR) < 0.1** | |
| percentage Asian |  |
| median rent |  |
| percentage urban |  |
| Supplemental Nutrition Assistance Program (SNAP) |  |
| **Other sociodemographic variables with abs(correlation with UVR) ≥ 0.1 [not used for most analyses]** | |
| percentage colon cancer screened |  |
| percentage white |  |
| percentage black |  |
| percentage Hispanic |  |
| income per capita |  |
| low income food desert |  |
| poverty rate |  |
| food desert |  |
| percentage diabetic |  |
| percentage obese |  |

**Table A4. Absolute incidence rates of malignant and non-malignant brain and CNS tumour by major histopathological subtype and age group**

| Tumour subtype | Age 0-5 y | |  | Age 6-9 y | |  | Age 10-15 y | |  | Age 16-19 y | |  |
| --- | --- | --- | --- | --- | --- | --- | --- | --- | --- | --- | --- | --- |
|  | Cases | Incidence rate / 10^5^ person /year (95% CI) |  | Cases | Incidence rate / 10^5^ person /year (95% CI) |  | Cases | Incidence rate / 10^5^ person /year (95% CI) |  | Cases | Incidence rate / 10^5^ person /year (95% CI) | *p*-value heterogeneity |
| Malignant brain tumours | | | | | | | | | | | |  |
| Diffuse astrocytic and oligodendroglial tumours | 1186 | 0.467 (0.446, 0.489) |  | 852 | 0.497 (0.470, 0.525) |  | 1513 | 0.564 (0.541, 0.588) |  | 1221 | 0.671 (0.640, 0.702) | <0.0001 |
| Pilocytic astrocytoma | 2774 | 1.093 (1.056, 1.130) |  | 1641 | 0.958 (0.916, 1.001) |  | 2161 | 0.806 (0.775, 0.837) |  | 949 | 0.521 (0.491, 0.552) | <0.0001 |
| Other astrocytoma variants | 31 | 0.012 (0.010, 0.015) |  | 83 | 0.048 (0.042, 0.056) |  | 161 | 0.060 (0.054, 0.067) |  | 133 | 0.073 (0.065, 0.082) | <0.0001 |
| Ependymal tumours | 1019 | 0.401 (0.375, 0.429) |  | 372 | 0.217 (0.194, 0.242) |  | 437 | 0.163 (0.147, 0.180) |  | 262 | 0.144 (0.126, 0.164) | <0.0001 |
| Other gliomas | 2236 | 0.881 (0.850, 0.912) |  | 1414 | 0.826 (0.789, 0.863) |  | 1386 | 0.517 (0.494, 0.540) |  | 581 | 0.319 (0.297, 0.342) | <0.0001 |
| Tumours of the pineal region | 132 | 0.052 (0.044, 0.061) |  | 57 | 0.033 (0.026, 0.042) |  | 87 | 0.032 (0.026, 0.039) |  | 65 | 0.036 (0.028, 0.045) | 0.0007 |
| Embryonal tumours | 2710 | 1.067 (1.034, 1.101) |  | 1210 | 0.706 (0.673, 0.740) |  | 907 | 0.338 (0.320, 0.357) |  | 373 | 0.205 (0.188, 0.223) | <0.0001 |
| Tumours of meninges | 113 | 0.045 (0.038, 0.052) |  | 54 | 0.032 (0.025, 0.040) |  | 96 | 0.036 (0.030, 0.043) |  | 70 | 0.038 (0.031, 0.047) | 0.1007 |
| Non-malignant brain tumours | | | | | | | | | | | | |
| Other astrocytoma variants | 180 | 0.071 (0.059, 0.084) |  | 121 | 0.071 (0.057, 0.086) |  | 139 | 0.052 (0.042, 0.063) |  | 64 | 0.035 (0.026, 0.046) | <0.0001 |
| Ependymal tumours | 33 | 0.013 (0.010, 0.017) |  | 47 | 0.027 (0.021, 0.035) |  | 148 | 0.055 (0.048, 0.063) |  | 135 | 0.074 (0.064, 0.086) | <0.0001 |
| Neuronal and mixed neuronal-glial tumours | 603 | 0.237 (0.222, 0.254) |  | 456 | 0.266 (0.246, 0.288) |  | 1119 | 0.417 (0.397, 0.439) |  | 713 | 0.392 (0.367, 0.417) | <0.0001 |
| Choroid plexus tumours | 318 | 0.125 (0.113, 0.138) |  | 59 | 0.034 (0.027, 0.043) |  | 88 | 0.033 (0.027, 0.039) |  | 69 | 0.038 (0.030, 0.047) | <0.0001 |
| Tumours of cranial and paraspinal nerves | 444 | 0.175 (0.161, 0.190) |  | 272 | 0.159 (0.143, 0.176) |  | 550 | 0.205 (0.190, 0.221) |  | 552 | 0.303 (0.282, 0.326) | <0.0001 |
| Tumours of meninges | 690 | 0.272 (0.249, 0.296) |  | 200 | 0.117 (0.099, 0.136) |  | 598 | 0.223 (0.203, 0.244) |  | 723 | 0.397 (0.365, 0.431) | <0.0001 |
| Tumours of sellar region (including pituitary, craniopharyngioma) | 497 | 0.196 (0.176, 0.217) |  | 890 | 0.520 (0.480, 0.561) |  | 2446 | 0.912 (0.870, 0.955) |  | 4055 | 2.227 (2.148, 2.309) | <0.0001 |

**Table A5. Absolute incidence rates of malignant and non-malignant brain and CNS tumour by major histopathological subtype and racial/ethnic group**

| Tumour subtype | White non-Hispanic | |  | Black non-Hispanic | |  | Hispanic | |  | Asian/Pacific Islander | |  |
| --- | --- | --- | --- | --- | --- | --- | --- | --- | --- | --- | --- | --- |
|  | Cases | Incidence rate / 10^5^ person /year (95% CI) |  | Cases | Incidence rate / 10^5^ person /year (95% CI) |  | Cases | Incidence rate / 10^5^ person /year (95% CI) |  | Cases | Incidence rate / 10^5^ person /year (95% CI) | *p*-value heterogeneity |
| Malignant brain tumours | | | | | | | | | | | |  |
| Diffuse astrocytic and oligodendroglial tumours | 2755 | 0.659 (0.639, 0.680) |  | 569 | 0.475 (0.443, 0.509) |  | 1161 | 0.420 (0.400, 0.441) |  | 287 | 0.469 (0.425, 0.516) | <0.0001 |
| Pilocytic astrocytoma | 4679 | 1.119 (1.088, 1.151) |  | 773 | 0.645 (0.601, 0.691) |  | 1758 | 0.636 (0.607, 0.666) |  | 315 | 0.514 (0.460, 0.573) | <0.0001 |
| Other astrocytoma variants | 205 | 0.049 (0.044, 0.054) |  | 65 | 0.054 (0.045, 0.064) |  | 126 | 0.046 (0.040, 0.052) |  | 12 | 0.020 (0.013, 0.029) | <0.0001 |
| Ependymal tumours | 1062 | 0.254 (0.238, 0.271) |  | 238 | 0.199 (0.173, 0.227) |  | 674 | 0.244 (0.225, 0.264) |  | 116 | 0.189 (0.155, 0.229) | 0.0007 |
| Other gliomas | 3261 | 0.780 (0.756, 0.804) |  | 646 | 0.539 (0.503, 0.577) |  | 1418 | 0.513 (0.490, 0.538) |  | 292 | 0.477 (0.429, 0.528) | <0.0001 |
| Tumours of the pineal region | 157 | 0.038 (0.033, 0.043) |  | 87 | 0.073 (0.060, 0.087) |  | 82 | 0.030 (0.024, 0.036) |  | 15 | 0.024 (0.015, 0.037) | <0.0001 |
| Embryonal tumours | 2788 | 0.667 (0.646, 0.689) |  | 516 | 0.431 (0.399, 0.464) |  | 1552 | 0.562 (0.538, 0.586) |  | 344 | 0.562 (0.512, 0.615) | <0.0001 |
| Tumours of meninges | 166 | 0.040 (0.034, 0.046) |  | 40 | 0.033 (0.025, 0.044) |  | 103 | 0.037 (0.031, 0.044) |  | 24 | 0.039 (0.026, 0.056) | 0.7432 |
| Non-malignant brain tumours | | | | | | | | | | | | |
| Other astrocytoma variants | 240 | 0.057 (0.049, 0.067) |  | 80 | 0.067 (0.050, 0.087) |  | 148 | 0.054 (0.044, 0.065) |  | 36 | 0.059 (0.038, 0.086) | 0.6582 |
| Ependymal tumours | 228 | 0.055 (0.048, 0.061) |  | 15 | 0.013 (0.008, 0.019) |  | 98 | 0.035 (0.029, 0.042) |  | 22 | 0.036 (0.024, 0.052) | <0.0001 |
| Neuronal and mixed neuronal-glial tumours | 1750 | 0.419 (0.401, 0.436) |  | 321 | 0.268 (0.243, 0.295) |  | 661 | 0.239 (0.223, 0.256) |  | 159 | 0.260 (0.225, 0.298) | <0.0001 |
| Choroid plexus tumours | 294 | 0.070 (0.063, 0.078) |  | 52 | 0.043 (0.033, 0.056) |  | 164 | 0.059 (0.051, 0.068) |  | 24 | 0.039 (0.026, 0.056) | 0.0002 |
| Tumours of cranial and paraspinal nerves | 1032 | 0.247 (0.233, 0.261) |  | 188 | 0.157 (0.137, 0.178) |  | 487 | 0.176 (0.162, 0.191) |  | 111 | 0.181 (0.152, 0.214) | <0.0001 |
| Tumours of meninges | 1147 | 0.274 (0.257, 0.293) |  | 256 | 0.214 (0.185, 0.245) |  | 676 | 0.245 (0.224, 0.266) |  | 132 | 0.216 (0.177, 0.260) | 0.0017 |
| Tumours of sellar region (including pituitary, craniopharyngioma) | 3618 | 0.865 (0.835, 0.897) |  | 1051 | 0.877 (0.821, 0.936) |  | 2782 | 1.007 (0.967, 1.048) |  | 437 | 0.714 (0.643, 0.789) | <0.0001 |

**Table A6. Absolute incidence rates of malignant and non-malignant brain and CNS tumour by major histopathological subtype and sex**

| Tumour subtype | Male | |  | Female | | *p*-value heterogeneity |
| --- | --- | --- | --- | --- | --- | --- |
|  | Cases | Incidence rate / 10^5^ person /year (95% CI) |  | Cases | Incidence rate / 10^5^ person /year (95% CI) |  |
| Malignant brain tumours | | | | | |  |
| Diffuse astrocytic and oligodendroglial tumours | 2589 | 0.578 (0.560, 0.596) |  | 2183 | 0.511 (0.493, 0.529) | <0.0001 |
| Pilocytic astrocytoma | 3903 | 0.871 (0.846, 0.897) |  | 3622 | 0.848 (0.822, 0.873) | 0.1973 |
| Other astrocytoma variants | 219 | 0.049 (0.044, 0.054) |  | 189 | 0.044 (0.040, 0.049) | 0.1538 |
| Ependymal tumours | 1138 | 0.254 (0.239, 0.270) |  | 952 | 0.223 (0.208, 0.238) | 0.0041 |
| Other gliomas | 2707 | 0.604 (0.585, 0.624) |  | 2910 | 0.681 (0.660, 0.703) | <0.0001 |
| Tumours of the pineal region | 180 | 0.040 (0.035, 0.046) |  | 161 | 0.038 (0.032, 0.044) | 0.5384 |
| Embryonal tumours | 3074 | 0.686 (0.666, 0.707) |  | 2126 | 0.497 (0.480, 0.516) | <0.0001 |
| Tumours of meninges | 185 | 0.041 (0.036, 0.047) |  | 148 | 0.035 (0.030, 0.040) | 0.0806 |
| Non-malignant brain tumours | | | | | |  |
| Other astrocytoma variants | 286 | 0.064 (0.055, 0.074) |  |  | 0.051 (0.043, 0.060) | 0.0491 |
| Ependymal tumours | 223 | 0.050 (0.045, 0.055) |  | 140 | 0.033 (0.029, 0.037) | <0.0001 |
| Neuronal and mixed neuronal-glial tumours | 1604 | 0.358 (0.343, 0.373) |  | 1287 | 0.301 (0.287, 0.316) | <0.0001 |
| Choroid plexus tumours | 308 | 0.069 (0.062, 0.076) |  | 226 | 0.053 (0.047, 0.059) | 0.0011 |
| Tumours of cranial and paraspinal nerves | 930 | 0.208 (0.196, 0.220) |  | 888 | 0.208 (0.196, 0.220) | 0.9788 |
| Tumours of meninges | 1073 | 0.239 (0.224, 0.255) |  | 1138 | 0.266 (0.250, 0.283) | 0.0216 |
| Tumours of sellar region (including pituitary, craniopharyngioma) | 2337 | 0.522 (0.501, 0.543) |  | 5551 | 1.299 (1.265, 1.333) | <0.0001 |

**Table A7. Relative risks of malignant brain and CNS tumour in relation to solar ultraviolet cumulative radiant exposure.** Unless otherwise stated all CI are profile-likelihood based.

| Subgroup | Cases / Population-years | Fully-adjusted | | Adjusted without demographic/socioeconomic variables | |
| --- | --- | --- | --- | --- | --- |
|  |  | Relative risk / MJ cm^-2^ (+ 95% CI) | *p*-value | Relative risk / MJ cm^-2^ (+ 95% CI) | *p*-value |
| White non-Hispanic, male^a^ | 9022 / 214,572,499 | 0.529 (0.269, 1.083) | 0.0813 | 0.551 (0.284, 1.068) | 0.0774 |
| White non-Hispanic, female^a^ | 7389 / 203,465,614 | 0.169 (0.073^b^, 0.393^b^) | <0.0001 | 0.149 (0.069, 0.324) | <0.0001 |
| Black non-Hispanic, male^a^ | 1700 / 60,890,268 | 0.589 (0.097, 3.523) | 0.5628 | 0.563 (0.099, 3.168) | 0.5154 |
| Black non-Hispanic, female^a^ | 1545 / 58,913,842 | 1.324 (0.193^b^, 9.093^b^) | 0.7754 | 0.844 (0.141, 5.022) | 0.8528 |
| Hispanic, male^a^ | 4179 / 141,287,849 | 0.482 (0.178^b^, 1.305^b^) | 0.1514 | 0.323 (0.128, 0.819) | 0.0174 |
| Hispanic, female^a^ | 3561 / 135,012,220 | 0.289 (0.103, 0.845) | 0.0236 | 0.273 (0.097, 0.774) | 0.0146 |
| Asian/ Pacific Islander, male^a^ | 933 / 31,291,975 | 2.162 (0.455, 10.329) | 0.3325 | 1.343 (0.349, 5.209) | 0.6684 |
| Asian/ Pacific Islander, female^a^ | 759 / 29,945,368 | 0.270 (0.019, 3.870) | 0.3343 | 0.171 (0.016, 1.819) | 0.1426 |
| **Total (white+black non-Hispanic, Hispanic, Asian/ Pacific Islanders)^c^** | 29,088 / 875,379,635 | 0.357 (0.242^b^, 0.526^b^) | <0.0001 | 0.314 (0.219, 0.450) | <0.0001 |
|  |  |  |  |  |  |
|  |  |  |  |  |  |
|  |  |  |  |  |  |

^a^fully-adjusted models for age (10 group factor variable), percentage urban, percentage screened and Supplemental Nutrition Assistance Program (SNAP) in that order; models without demographic/socioeconomic adjustments adjust only for age.

^b^Wald-based CI.

^c^fully-adjusted models for age (10 group factor variable), racial/ethnic group (4 group factor variable), sex, percentage urban, percentage screened, Supplemental Nutrition Assistance Program (SNAP) and age x sex in that order; models without demographic/socioeconomic adjustments adjust only for age, racial/ethnic group, sex, age x sex.

**Table A8. Relative risks of non-malignant brain and CNS tumour in relation to solar ultraviolet cumulative radiant exposure.** Unless otherwise stated all CI are profile-likelihood based.

| Subgroup | Cases / Population-years | Fully-adjusted | | Adjusted without demographic/socioeconomic variables | |
| --- | --- | --- | --- | --- | --- |
|  |  | Relative risk / MJ cm^-2^ (+ 95% CI) | *p*-value | Relative risk / MJ cm^-2^ (+ 95% CI) | *p*-value |
| White non-Hispanic, male^a^ | 4298 / 214,572,499 | 0.177 (0.078, 0.405) | <0.0001 | 0.168 (0.075, 0.372) | <0.0001 |
| White non-Hispanic, female^a^ | 5334 / 203,465,614 | 0.252 (0.114^b^, 0.556^b^) | 0.0006 | 0.231 (0.110, 0.483) | <0.0001 |
| Black non-Hispanic, male^a^ | 920 / 60,890,268 | 0.434 (0.049, 3.777) | 0.4507 | 0.403 (0.049, 3.271) | 0.3960 |
| Black non-Hispanic, female^a^ | 1300 / 58,913,842 | 0.253 (0.062, 1.026) | 0.0544 | 0.138 (0.038, 0.505) | 0.0027 |
| Hispanic, male^a^ | 2310 / 141,287,849 | 0.174 (0.048^b^, 0.632^b^) | 0.0081 | 0.107 (0.035, 0.332) | 0.0001 |
| Hispanic, female^a^ | 3419 / 135,012,220 | 0.067 (0.032, 0.139) | <0.0001 | 0.048 (0.024, 0.094) | <0.0001 |
| Asian/ Pacific Islander, male^a^ | 449 / 31,291,975 | 0.028 (0.003, 0.279) | 0.0024 | 0.050 (0.006, 0.426) | 0.0061 |
| Asian/ Pacific Islander, female^a^ | 555 / 29,945,368 | 0.006 (0.000, 0.207) | 0.0048 | 0.006 (0.000, 0.401) | 0.0170 |
| **Total (white+black non-Hispanic, Hispanic, Asian/ Pacific Islanders)^c^** | 18,585 / 875,379,635 | 0.148 (0.095, 0.232) | <0.0001 | 0.122 (0.079, 0.190) | <0.0001 |
|  |  |  |  |  |  |
|  |  |  |  |  |  |
|  |  |  |  |  |  |

^a^fully-adjusted models for age (10 group factor variable), calendar year, Supplemental Nutrition Assistance Program (SNAP), median rent, percentage Asian, percentage screened, percentage urban in that order; models without demographic/socioeconomic adjustments adjust only for age and calendar year.

^b^Wald-based CI.

^c^fully-adjusted models for age (10 group factor variable), calendar year, sex, racial/ethnic group (4 group factor variable), Supplemental Nutrition Assistance Program (SNAP), median rent, percentage Asian, percentage screened, percentage urban, age x sex, racial/ethnic group x sex, year x race in that order; models without demographic/socioeconomic adjustments adjust only for age, calendar year, sex, racial/ethnic group, age x sex, racial/ethnic group x sex, year x race.

**Table A9. Malignant and non-malignant brain and CNS tumours cumulative UVR radiant exposure and UVR irradiance relative risks for potentially problematic subtypes, as well as those with under 300 cases.** Tumours are coded according to the classification given in Table A1. All relative risks are derived using a model adjusted for age (10-level factor), racial/ethnic group (4-level factor), calendar year and sex. Unless otherwise stated all CI are derived from the profile likelihood. *p*-values for heterogeneity are derived via fitting a restricted-maximum likelihood model to the ln[RR] by endpoint.

| Tumour subtype | Cases | Cumulative radiant exposure | |  | Irradiance | | *p*-value heterogeneity [cumulative radiant exposure / irradiance] |
| --- | --- | --- | --- | --- | --- | --- | --- |
|  |  | Relative risk / MJ cm^-2^ (+ 95% CI) | *p*-value |  | Relative risk / mW cm^-2^ (+ 95% CI) | *p*-value |  |
| Malignant brain tumours | | | | | | |  |
| Neuronal and mixed neuronal-glial tumours | 225 | 0.109 (0.008, 1.417) | 0.0905 |  | 0.519 (0.196, 1.367) | 0.1847 | <0.0001 / 0.0026 |
| Choroid plexus tumours | 190 | 0.009 (0.000, 4.771) | 0.1408 |  | 0.800 (0.235, 2.718) | 0.7208 |  |
| Tumours of cranial and paraspinal nerves | 52 | 6.709 (0.000, >1000) | <0.0001 |  | 7.726 (0.127, 497.1) | <0.0001 |  |
| Tumours of meninges | 333 | 0.373 (0.019, 7.269) | <0.0001 |  | 0.726 (0.240, 2.193) | <0.0001 |  |
| Lymphoma | 290 | 4.745 (0.312, 72.64) | 0.2623 |  | 2.296 (0.734, 7.196) | 0.1530 |  |
| Other haematopoietic neoplasms | 3 | 0.000 (0.000^a^, 0.004^a^) | <0.0001 |  | 0.208 (0.081, 0.532) | 0.0009 |  |
| Germ cell tumours | 1622 | 2.647 (0.721, 9.736) | 0.1426 |  | 1.697 (0.966, 2.983) | 0.0660 |  |
| Tumours of sellar region (including pituitary, craniopharyngioma) | 14 | 0.000 (0.000^a^, 216.9^a^) | 0.2318 |  | 0.022 (0.000, 17.69) | 0.2559 |  |
| Haemangioma, other and unclassified tumours | 406 | 0.854 (0.003, 251.7) | 0.9566 |  | 1.086 (0.135, 8.659) | 0.9382 |  |
| Non-malignant brain tumours | | | | | | |  |
| Diffuse astrocytic and oligodendroglial tumours | 3 | >1000 (1.130, >1000) | 0.0475 |  | >1000 (122.1, >1000) | <0.0001 | <0.0001 / <0.0001 |
| Other gliomas | 10 | 0.000 (0.000^a^, 17.30^a^) | 0.1238 |  | 0.143 (0.003^a^, 6.109^a^) | 0.3044 |  |
| Tumours of the pineal region | 60 | 15.241 (0.715^a^, 324.9^a^) | 0.0814 |  | 4.581 (1.131, 18.53) | 0.0330 |  |
| Lymphoma | 15 | 436.1 (0.003^a^, >1000^a^) | <0.0001 |  | 98.65 (0.798^a^, >1000^a^) | .0.0565 |  |
| Other haematopoietic neoplasms | 3 | 0.000 (0.000^a^, 0.000^a^) | <0.0001 |  | 0.007 (0.002^a^, 0.027^a^) | <0.0001 |  |
| Germ cell tumours | 133 | 0.184 (0.001, 40.663) | 0.5397 |  | 0.302 (0.057, 1.600) | 0.1596 |  |
| Haemangioma, other and unclassified tumours | 2152 | 1.006 (0.247, 4.084) | 0.9933 |  | 1.061 (0.597, 1.885) | 0.8387 |  |

^a^using Wald-based CI

**Table A10. Significance of modification of relative risk by racial/ethnic group and sex for malignant and non-malignant brain and CNS tumour.**

| Model type | *p*-value for modification of relative risk by race | |  | *p*-value for modification of relative risk by sex | |
| --- | --- | --- | --- | --- | --- |
|  | Cumulative radiant exposure | Irradiance |  | Cumulative radiant exposure | Irradiance |
| Malignant brain and CNS tumour | | | | | |
| Fully-adjusted model^a^ | 0.0008 | 0.4492 |  | 0.0254 | 0.1201 |
| Model without demographic/socioeconomic adjustments^b^ | 0.0008 | 0.4718 |  | 0.0220 | 0.1132 |
| Non-malignant brain and CNS tumour | | | | | |
| Fully-adjusted model^c^ | 0.0462 | 0.0208 |  | 0.5299 | 0.3961 |
| Model without demographic/socioeconomic adjustments^d^ | 0.0418 | 0.0152 |  | 0.5341 | 0.3893 |

^a^adjusted for age (10 group factor variable), racial/ethnic group (4 group factor variable), sex, percentage urban, percentage screened, Supplemental Nutrition Assistance Program (SNAP) and age x sex in that order

^b^adjusted for age, racial/ethnic group, sex, age x sex.

^c^adjusted for age (10 group factor variable), calendar year, sex, racial/ethnic group (4 group factor variable), Supplemental Nutrition Assistance Program (SNAP), median rent, percentage Asian, percentage screened, percentage urban, age x sex, racial/ethnic group x sex, year x race in that order

^d^adjusted for age, calendar year, sex, racial/ethnic group, age x sex, racial/ethnic group x sex, year x race.

**Table A11. Relative risks of malignant and non-malignant brain and CNS tumour by racial/ethnic group.** Unless otherwise stated all CI are profile-likelihood based.

|  | Cumulative radiant exposure | |  | Irradiance | |
| --- | --- | --- | --- | --- | --- |
| Racial/ethnic group | Relative risk / MJ cm^-2^ (+ 95% CI) | Heterogeneity *p*-value |  | Relative risk / mW cm^-2^ (+ 95% CI) | Heterogeneity *p*-value |
|  |  |  |  |  |  |
|  |  |  |  |  |  |
| Malignant brain and CNS tumour^a^ | | | | | |
| White non-Hispanic | 0.424 (0.282, 0.638) | 0.0008 |  | 0.717 (0.601^b^, 0.855^b^) | 0.4492 |
| Black non-Hispanic | 0.464 (0.292, 0.738) |  |  | 1.036 (0.679^b^, 1.582^b^) |  |
| Hispanic | 0.309 (0.208, 0.458) |  |  | 0.762 (0.604^b^, 0.962^b^) |  |
| Asian + Pacific Islander | 0.546 (0.331, 0.898) |  |  | 0.691 (0.426^b^, 1.122^b^) |  |
| Non-malignant brain and CNS tumour^c^ | | | | | |
| White non-Hispanic | 0.139 (0.087, 0.223) | 0.0462 |  | 0.590 (0.444, 0.784) | 0.0208 |
| Black non-Hispanic | 0.154 (0.087, 0.273) |  |  | 0.586 (0.301, 1.135) |  |
| Hispanic | 0.165 (0.105, 0.259) |  |  | 0.367 (0.262, 0.513) |  |
| Asian + Pacific Islander | 0.076 (0.040, 0.147) |  |  | 0.196 (0.089, 0.432) |  |

^a^adjusted for age (10 group factor variable), racial/ethnic group (4 group factor variable), sex, percentage urban, percentage screened, Supplemental Nutrition Assistance Program (SNAP) and age x sex in that order.

^b^Wald-based CI.

^c^adjusted for age (10 group factor variable), calendar year, sex, racial/ethnic group (4 group factor variable), Supplemental Nutrition Assistance Program (SNAP), median rent, percentage Asian, percentage screened, percentage urban, age x sex, racial/ethnic group x sex, year x race in that order.

**Table A12. Relative risks of malignant and non-malignant brain and CNS tumour by sex.** Unless otherwise stated all CI are profile-likelihood based.

| Sex | Cumulative radiant exposure | |  | Irradiance | |
| --- | --- | --- | --- | --- | --- |
|  | Relative risk / MJ cm^-2^ (+ 95% CI) | Heterogeneity *p*-value |  | Relative risk / mW cm^-2^ (+ 95% CI) | Heterogeneity *p*-value |
| Malignant brain and CNS tumour^a^ | | | | | |
| Females | 0.224 (0.127^b^, 0.393^b^) | 0.0254 |  | 0.680 (0.564^b^, 0.820^b^) | 0.1201 |
| Males | 0.506 (0.309^b^, 0.829^b^) |  |  | 0.822 (0.691^b^, 0.976^b^) |  |
| Non-malignant brain and CNS tumour^c^ | | | | | |
| Females | 0.133 (0.076, 0.233) | 0.5299 |  | 0.433 (0.333, 0.561) | 0.3961 |
| Males | 0.178 (0.087, 0.364) |  |  | 0.513 (0.380, 0.693) |  |

^a^adjusted for age (10 group factor variable), racial/ethnic group (4 group factor variable), sex, percentage urban, percentage screened, Supplemental Nutrition Assistance Program (SNAP) and age x sex in that order.

^b^Wald-based CI.

^c^adjusted for age (10 group factor variable), calendar year, sex, racial/ethnic group (4 group factor variable), Supplemental Nutrition Assistance Program (SNAP), median rent, percentage Asian, percentage screened, percentage urban, age x sex, racial/ethnic group x sex, year x race in that order.

**Table A13. Relative risks of** **malignant and non-malignant brain and CNS tumours by age, using either UVR cumulative radiant exposure (MJ cm^-2^) or UVR irradiance (mW cm^-2^)**.

| Attained age (years) | Cases / Population | Ultraviolet cumulative radiant exposure | | | |  | Ultraviolet irradiance | | | |
| --- | --- | --- | --- | --- | --- | --- | --- | --- | --- | --- |
|  |  | Fully-adjusted | | Adjusted without demographic/socioeconomic variables | |  | Fully-adjusted | | Adjusted without demographic/socioeconomic variables | |
|  |  | Relative risk / MJ cm^-2^ (+ 95% CI) | *p*-value | Relative risk / MJ cm^2^ (+ 95% CI) | *p*-value |  | Relative risk / mW cm^-2^ (+ 95% CI) | *p*-value | Relative risk / mW cm^-2^ (+ 95% CI) | *p*-value |
| Malignant Brain and CNS^a^ | | | | | | | | | | |
| 0-3 | 7348 / 168,729,394 | 0.082 (0.003^b^, 2.456^b^) | 0.7885 | 0.044 (0.002, 1.177) | 0.7202 |  | 0.913 (0.715^b^, 1.166^b^) | 0.0984 | 0.866 (0.685, 1.096) | 0.0824 |
| 4-5 | 3457 / 85,172,850 | 0.218 (0.024^b^, 2.002^b^) |  | 0.159 (0.018, 1.377) |  |  | 0.790 (0.556^b^, 1.121^b^) |  | 0.749 (0.533, 1.053) |  |
| 6-7 | 3229 / 85,282,068 | 1.082 (0.211^b^, 5.551^b^) |  | 0.866 (0.176, 4.251) |  |  | 1.022 (0.712^b^, 1.467^b^) |  | 0.970 (0.682, 1.379) |  |
| 8-9 | 2862 / 86,007,744 | 0.275 (0.071^b^, 1.059^b^) |  | 0.231 (0.062, 0.858) |  |  | 0.696 (0.474^b^, 1.021^b^) |  | 0.660 (0.454, 0.959) |  |
| 10-11 | 2701 / 88,858,151 | 0.280 (0.090^b^, 0.871^b^) |  | 0.243 (0.080, 0.732) |  |  | 0.646 (0.435^b^, 0.958^b^) |  | 0.612 (0.417, 0.899) |  |
| 12-13 | 2591 / 89,821,293 | 0.527 (0.198^b^, 1.400^b^) |  | 0.467 (0.180, 1.209) |  |  | 0.772 (0.517^b^, 1.153^b^) |  | 0.733 (0.495, 1.083) |  |
| 14-15 | 2537 / 89,444,142 | 0.441 (0.187^b^, 1.037^b^) |  | 0.397 (0.172, 0.913) |  |  | 0.682 (0.454^b^, 1.022^b^) |  | 0.646 (0.435, 0.959) |  |
| 16-17 | 2350 / 90,173,924 | 0.352 (0.161^b^, 0.771^b^) |  | 0.321 (0.149, 0.688) |  |  | 0.574 (0.377^b^, 0.874^b^) |  | 0.544 (0.361, 0.820) |  |
| 18-19 | 2013 / 91,890,069 | 0.256 (0.120^b^, 0.547^b^) |  | 0.235 (0.112, 0.492) |  |  | 0.444 (0.282^b^, 0.700^b^) |  | 0.420 (0.269, 0.654) |  |
| Non-Malignant Brain and CNS^c^ | | | | | | | | | | |
| 0-3 | 2412 / 168,729,394 | 0.768 (0.000, >1000) | 0.3820 | 0.195 (0.000, 770.5) | 0.3348 |  | 1.045 (0.625, 1.746) | 0.0075 | 0.947 (0.565, 1.584) | 0.0082 |
| 4-5 | 951 / 85,172,850 | 0.121 (0.001, 20.83) |  | 0.066 (0.000, 11.69) |  |  | 0.717 (0.317, 1.616) |  | 0.652 (0.287, 1.475) |  |
| 6-7 | 1092 / 85,282,068 | 0.235 (0.008, 7.272) |  | 0.154 (0.005, 4.848) |  |  | 0.727 (0.340, 1.552) |  | 0.662 (0.308, 1.418) |  |
| 8-9 | 1295 / 86,007,744 | 0.005 (0.000, 0.065) |  | 0.004 (0.000, 0.046) |  |  | 0.228 (0.113, 0.460) |  | 0.206 (0.102, 0.417) |  |
| 10-11 | 1415 / 88,858,151 | 0.253 (0.037, 1.726) |  | 0.195 (0.028, 1.340) |  |  | 0.621 (0.318, 1.210) |  | 0.567 (0.290, 1.107) |  |
| 12-13 | 1796 / 89,821,293 | 0.083 (0.019, 0.354) |  | 0.066 (0.015, 0.283) |  |  | 0.361 (0.199, 0.654) |  | 0.328 (0.180, 0.596) |  |
| 14-15 | 2647 / 89,444,142 | 0.182 (0.064, 0.512) |  | 0.150 (0.053, 0.424) |  |  | 0.447 (0.273, 0.729) |  | 0.408 (0.249, 0.666) |  |
| 16-17 | 3406 / 90,173,924 | 0.188 (0.084, 0.420) |  | 0.158 (0.070, 0.355) |  |  | 0.408 (0.264, 0.629) |  | 0.372 (0.241, 0.574) |  |
| 18-19 | 3571 / 91,890,069 | 0.151 (0.074, 0.306) |  | 0.129 (0.064, 0.262) |  |  | 0.323 (0.211, 0.492) |  | 0.293 (0.192, 0.448) |  |

^a^fully-adjusted models for age (10 group factor variable), racial/ethnic group (4 group factor variable), sex, percentage urban, percentage screened, Supplemental Nutrition Assistance Program (SNAP) and age x sex in that order; models without demographic/socioeconomic adjustments adjust only for age, racial/ethnic group, sex, age x sex.

^b^Wald-based CI.

^c^fully-adjusted models for age (10 group factor variable), calendar year, sex, racial/ethnic group (4 group factor variable), Supplemental Nutrition Assistance Program (SNAP), median rent, percentage Asian, percentage screened, percentage urban, age x sex, racial/ethnic group x sex, year x race in that order; models without demographic/socioeconomic adjustments adjust only for age, calendar year, sex, racial/ethnic group, age x sex, racial/ethnic group x sex, year x race.

**Table A14. Relative risks of malignant and non-malignant brain and CNS tumour, using all available background variables, with no exclusions for those with UV correlations >0.1. Risks are for total (white+black non-Hispanic, Hispanic, Asian/ Pacific Islanders).** Unless otherwise stated all CI are derived from the profile likelihood.

| Endpoint | Cases / Population-years | Ultraviolet cumulative radiant exposure | |  | Ultraviolet irradiance | |
| --- | --- | --- | --- | --- | --- | --- |
|  |  | Relative risk / MJ cm^-2^ (+ 95% CI) | *p*-value |  | Relative risk / mW cm^-2^ (+ 95% CI) | *p*-value |
| Malignant brain and CNS tumour^a^ | 29,088 / 875,379,635 | 0.477 (0.307, 0.742) | 0.0010 |  | 0.879 (0.744, 1.039) | 0.1298 |
| Non-malignant brain and CNS tumour^b^ | 18,585 / 875,379,635 | 0.183 (0.097, 0.345) | <0.0001 |  | 0.526 (0.384, 0.721) | <0.0001 |

^a^adjusted for age (10 group factor variable), racial/ethnic group (4 group factor variable), sex, calendar year, income per capita, urban, percentage White, percentage diabetic, percentage poverty, Supplemental Nutrition Assistance Program (SNAP), percentage Hispanic, food desert, age x sex, age x racial/ethnic group, year x , racial/ethnic group in that order.

^b^adjusted for age (10 group factor variable), calendar year, sex, racial/ethnic group (4 group factor variable), Supplemental Nutrition Assistance Program (SNAP), percentage poverty, percentage diabetic, low-income food desert, percentage Hispanic, median rent, percentage Asian, urban, food desert, percentage Black, percentage White, income per capita, age x sex, racial/ethnic group x sex, year x racial/ethnic group in that order.

**Table A15. Relative risks of malignant brain and CNS tumour in relation to either UVR cumulative radiant exposure (MJ cm^-2^) or UVR irradiance (mW cm^-2^) by median rent ^a^**. Unless otherwise stated all CI are profile-likelihood based.

| Median rent ($) | Cases | Cumulative radiant exposure | |  | Irradiance | |
| --- | --- | --- | --- | --- | --- | --- |
|  |  | Relative risk / MJ cm^-2^ (+ 95% CI) | *p*-value heterogeneity |  | Relative risk / mW cm^-2^ (+ 95c% CI) | *p*-value heterogeneity |
| <300 | 70 | 0.287 (0.104^b^, 0.792^b^) | 0.1126 |  | 0.811 (0.582^b^, 1.131^b^) | 0.0010 |
| 300-399 | 753 | 0.303 (0.183^b^, 0.501 ^b^) |  |  | 0.731 (0.611^b^, 0.874^b^) |  |
| 400-499 | 1802 | 0.265 (0.170^b^, 0.414^b^) |  |  | 0.707 (0.604^b^, 0.827^b^) |  |
| 500-599 | 2895 | 0.309 (0.201^b^, 0.475^b^) |  |  | 0.711 (0.613^b^, 0.826^b^) |  |
| 600-699 | 3227 | 0.368 (0.246^b^, 0.549^b^) |  |  | 0.767 (0.668^b^, 0.881^b^) |  |
| 700-799 | 4589 | 0.355 (0.239^b^, 0.527^b^) |  |  | 0.738 (0.643^b^, 0.847^b^) |  |
| 800+ | 15,752 | 0.321 (0.214^b^, 0.481^b^) |  |  | 0.671 (0.580^b^, 0.777^b^) |  |

^a^adjusted for age (10 group factor variable), racial/ethnic group (4 group factor variable), sex, percentage urban, percentage screened, Supplemental Nutrition Assistance Program (SNAP) and age x sex in that order.

^b^using Wald-based CI.

**Table A16. Relative risks of non-malignant brain and CNS tumour in relation to either UVR cumulative radiant exposure (MJ cm^-2^) or UVR irradiance (mW cm^-2^) by median rent^a^**. Unless otherwise stated all CI are profile-likelihood based.

| Median rent ($) | Cases | Cumulative radiant exposure | |  | Irradiance | |
| --- | --- | --- | --- | --- | --- | --- |
|  |  | Relative risk / MJ cm^-2^ (+ 95% CI) | *p*-value heterogeneity |  | Relative risk / mW cm^-2^ (+ 95% CI) | *p*-value heterogeneity |
| <300 | 41 | 0.174 (0.050, 0.533) | 0.9672 |  | 0.506 (0.290, 0.842) | 0.7917 |
| 300-399 | 467 | 0.139 (0.077, 0.250) |  |  | 0.493 (0.377, 0.643) |  |
| 400-499 | 1205 | 0.141 (0.085, 0.234) |  |  | 0.492 (0.392, 0.617) |  |
| 500-599 | 1849 | 0.134 (0.082, 0.220) |  |  | 0.476 (0.382, 0.592) |  |
| 600-699 | 2005 | 0.143 (0.090, 0.226) |  |  | 0.471 (0.384, 0.577) |  |
| 700-799 | 2815 | 0.152 (0.097, 0.240) |  |  | 0.464 (0.379, 0.568) |  |
| 800+ | 10,203 | 0.149 (0.093, 0.237) |  |  | 0.436 (0.353, 0.539) |  |

^c^adjusted for age (10 group factor variable), calendar year, sex, racial/ethnic group (4 group factor variable), Supplemental Nutrition Assistance Program (SNAP), median rent, percentage Asian, percentage screened, percentage urban, age x sex, racial/ethnic group x sex, year x race in that order.

**Table A17. Effect of omission of particular states on relative risks of malignant and non-malignant brain/CNS tumour in relation to either UVR cumulative radiant exposure (MJ cm^-2^) or UVR irradiance (mW cm^-2^)**

| Omitted state | Cases / Population | Relative risk / MJ cm^-2^ (+ 95% CI) | *p*-value |  | Relative risk / mW cm^-2^ (+ 95c% CI) | *p*-value |
| --- | --- | --- | --- | --- | --- | --- |
| Malignant brain/CNS tumour^a^ | | | | | | |
| California | 22,597 / 650,751,971 | 0.572 (0.352, 0.929) | 0.0239 |  | 1.008 (0.856, 1.189) | 0.9283 |
| Connecticut | 28,380 / 855,529,045 | 0.361 (0.244^b^, 0.534^b^) | <0.0001 |  | 0.757 (0.661^b^, 0.866^b^) | <0.0001 |
| Georgia | 27,197 / 815,882,203 | 0.377 (0.255^b^, 0.559^b^) | <0.0001 |  | 0.770 (0.671^b^, 0.883^b^) | 0.0002 |
| Iowa | 28,430 / 857,357,559 | 0.350 (0.235^b^, 0.520^b^) | <0.0001 |  | 0.752 (0.656^b^, 0.864^b^) | <0.0001 |
| Idaho | 28,766 / 865,200,564 | 0.347 (0.235^b^, 0.512^b^) | <0.0001 |  | 0.746 (0.651, 0.854) | <0.0001 |
| Illinois | 26,805 / 800,035,828 | 0.240 (0.162^b^, 0.356^b^) | <0.0001 |  | 0.643 (0.561^b^, 0.738^b^) | <0.0001 |
| Kentucky | 28,106 / 850,566,008 | 0.368 (0.251^b^, 0.539^b^) | <0.0001 |  | 0.762 (0.667^b^, 0.870^b^) | <0.0001 |
| Louisiana | 28,190 / 847,875,396 | 0.372 (0.253^b^, 0.547^b^) | <0.0001 |  | 0.766 (0.670^b^, 0.876^b^) | 0.0001 |
| Massachusetts | 27,823 / 839,558,275 | 0.338 (0.229^b^, 0.500^b^) | <0.0001 |  | 0.739 (0.646^b^, 0.845^b^) | <0.0001 |
| New Jersey | 27,245 / 825,100,492 | 0.394 (0.263^b^, 0.588^b^) | <0.0001 |  | 0.783 (0.681^b^, 0.901^b^) | 0.0006 |
| New Mexico | 28,836 / 864,944,650 | 0.404 (0.272^b^, 0.599^b^) | <0.0001 |  | 0.790 (0.689^b^, 0.906^b^) | 0.0007 |
| New York | 25,161 / 767,528,624 | 0.526 (0.341, 0.812) | 0.0037 |  | 0.875 (0.752, 1.019) | 0.0856 |
| Texas | 23,276 / 709,798,320 | 0.196 (0.128, 0.299) | <0.0001 |  | 0.557 (0.481, 0.647) | <0.0001 |
| Utah | 28,344 / 854,845,660 | 0.365 (0.246^b^, 0.539^b^) | <0.0001 |  | 0.763 (0.666^b^, 0.874^b^) | <0.0001 |
| Washington | 28,076 / 850,340,295 | 0.432 (0.286^b^, 0.653^b^) | <0.0001 |  | 0.801 (0.694^b^, 0.925^b^) | 0.0025 |
| None | 29,088 / 875,379,635 | 0.357 (0.242^b^, 0.526^b^) | <0.0001 |  | 0.754 (0.659^b^, 0.862^b^) | <0.0001 |
| Non-malignant brain/CNS tumour^c^ | | | | | | |
| California | 14,692 / 650,751,971 | 0.326 (0.176, 0.600) | 0.0003 |  | 0.818 (0.622, 1.075) | 0.1503 |
| Connecticut | 18,213 / 855,529,045 | 0.129 (0.082, 0.202) | <0.0001 |  | 0.438 (0.359, 0.535) | <0.0001 |
| Georgia | 17,354 / 815,882,203 | 0.149 (0.094, 0.234) | <0.0001 |  | 0.464 (0.378, 0.568) | <0.0001 |
| Iowa | 18,223 / 857,357,559 | 0.142 (0.090, 0.225) | <0.0001 |  | 0.455 (0.371, 0.558) | <0.0001 |
| Idaho | 18,438 / 865,200,564 | 0.148 (0.094, 0.231) | <0.0001 |  | 0.464 (0.380, 0.566) | <0.0001 |
| Illinois | 17,123 / 800,035,828 | 0.126 (0.079, 0.202) | <0.0001 |  | 0.417 (0.338, 0.515) | <0.0001 |
| Kentucky | 17,947 / 850,566,008 | 0.156 (0.098, 0.247) | <0.0001 |  | 0.477 (0.389, 0.586) | <0.0001 |
| Louisiana | 18,080 / 847,875,396 | 0.152 (0.097, 0.239) | <0.0001 |  | 0.471 (0.386, 0.575) | <0.0001 |
| Massachusetts | 17,900 / 839,558,275 | 0.114 (0.071^b^, 0.184^b^) | <0.0001 |  | 0.419 (0.339^b^, 0.519^b^) | <0.0001 |
| New Jersey | 17,395 / 825,100,492 | 0.183 (0.116, 0.291) | <0.0001 |  | 0.514 (0.419, 0.630) | <0.0001 |
| New Mexico | 18,435 / 864,944,650 | 0.166 (0.113, 0.245) | <0.0001 |  | 0.491 (0.414, 0.581) | <0.0001 |
| New York | 15,664 / 767,528,624 | 0.311 (0.181^b^, 0.535^b^) | <0.0001 |  | 0.725 (0.568^b^, 0.925^b^) | 0.0098 |
| Texas | 14,743 / 709,798,320 | 0.062 (0.036, 0.106) | <0.0001 |  | 0.280 (0.219, 0.358) | <0.0001 |
| Utah | 18,126 / 854,845,660 | 0.150 (0.095, 0.235) | <0.0001 |  | 0.471 (0.386, 0.576) | <0.0001 |
| Washington | 17,857 / 850,340,295 | 0.227 (0.141, 0.366) | <0.0001 |  | 0.535 (0.433, 0.662) | <0.0001 |
| None | 18,585 / 875,379,635 | 0.148 (0.095, 0.232) | <0.0001 |  | 0.466 (0.382, 0.567) | <0.0001 |

^a^adjusted for age (10 group factor variable), racial/ethnic group (4 group factor variable), sex, percentage urban, percentage screened, Supplemental Nutrition Assistance Program (SNAP) and age x sex in that order.

^b^Wald-based CI.

^c^adjusted for age (10 group factor variable), calendar year, sex, racial/ethnic group (4 group factor variable), Supplemental Nutrition Assistance Program (SNAP), median rent, percentage Asian, percentage screened, percentage urban, age x sex, racial/ethnic group x sex, year x race in that order.

**Table A18. Effect of omission of the follow-up in the period 2017-2021 on relative risks of malignant and non-malignant brain/CNS tumour in relation to either UVR cumulative radiant exposure (MJ cm^-2^) or UVR irradiance (mW cm^-2^)**

| Cases / Population | Relative risk / MJ cm^-2^ (+ 95% CI) | *p*-value |  | Relative risk / mW cm^-2^ (+ 95% CI) | *p*-value |
| --- | --- | --- | --- | --- | --- |
| Malignant brain/CNS tumour^a^ | | | | | |
| 22,884 / 675,820,869 | 0.383 (0.247^b^, 0.594^b^) | <0.0001 |  | 0.789 (0.678, 0.918) | 0.0022 |
| Non-malignant brain/CNS tumour^c^ | | | | | |
| 12,962 / 675,820,869 | 0.188 (0.106, 0.332) | <0.0001 |  | 0.501 (0.389, 0.645) | <0.0001 |

^a^adjusted for age (10 group factor variable), racial/ethnic group (4 group factor variable), sex, percentage urban, percentage screened, Supplemental Nutrition Assistance Program (SNAP) and age x sex in that order.

^b^Wald-based CI.

^c^adjusted for age (10 group factor variable), calendar year, sex, racial/ethnic group (4 group factor variable), Supplemental Nutrition Assistance Program (SNAP), median rent, percentage Asian, percentage screened, percentage urban, age x sex, racial/ethnic group x sex, year x race in that order.

**Table A19. Brain and CNS tumour relative risks in relation to either UVR cumulative radiant exposure (MJ cm^-2^) or UVR irradiance (mW cm^-2^) excluding certain potentially problematic subtypes**

| Omitted tumour endpoint | Cases | Relative risk / MJ cm^-2^ (+ 95% CI) | *p*-value |  | Relative risk / mW cm^-2^ (+ 95c% CI) | *p*-value |
| --- | --- | --- | --- | --- | --- | --- |
| Malignant brain/CNS tumour^a^ | | | | | | |
| None | 29,088 | 0.357 (0.242^b^, 0.526^b^) | <0.0001 |  | 0.754 (0.659^b^, 0.862^b^) | <0.0001 |
| Lymphoma, other haemopoietic, germ cell, haemangioma and other unclassified | 26,767 | 0.255 (0.170^b^, 0.382^b^) | <0.0001 |  | 0.708 (0.617^b^, 0.811^b^) | <0.0001 |
| Lymphoma, other haemopoietic, germ cell, haemangioma and other unclassified, sellar region | 26,518 | 0.403 (0.267, 0.608) | <0.0001 |  | 0.810 (0.702, 0.934) | 0.0037 |
| Non-malignant brain/CNS tumour^c^ | | | | | | |
| None | 18,585 | 0.148 (0.095, 0.232) | <0.0001 |  | 0.466 (0.382, 0.567) | <0.0001 |
| Lymphoma, other haemopoietic, germ cell, haemangioma and other unclassified | 16,282 | 0.114 (0.070, 0.183) | <0.0001 |  | 0.409 (0.329, 0.507) | <0.0001 |
| Lymphoma, other haemopoietic, germ cell, haemangioma and other unclassified, sellar region | 8394 | 0.205 (0.108^b^, 0.389^b^) | <0.0001 |  | 0.651 (0.501^b^, 0.847^b^) | 0.0013 |

^a^adjusted for age (10 group factor variable), racial/ethnic group (4 group factor variable), sex, percentage urban, percentage screened, Supplemental Nutrition Assistance Program (SNAP) and age x sex in that order.

^b^Wald-based CI.

^c^adjusted for age (10 group factor variable), calendar year, sex, racial/ethnic group (4 group factor variable), Supplemental Nutrition Assistance Program (SNAP), median rent, percentage Asian, percentage screened, percentage urban, age x sex, racial/ethnic group x sex, year x race in that order.

**References**

1. Surveillance Epidemiology and End Results (SEER) Program (www.seer.cancer.gov). SEER*Stat Database: Incidence – SEER Research Plus Limited-Field Data, 22 Registries, Nov 2022 Sub (2000-2020) - Linked To County Attributes - Total U.S., 1969-2020 Counties, National Cancer Institute, DCCPS, Surveillance Research Program, released April 2023, based on the November 2022 submission. National Cancer Institute, Bethesda, MD, USA. 2023. Accessed 9/2023.

2. Tatalovich Z, Wilson JP, Cockburn M. A comparison of Thiessen polygon, kriging, and spline models of potential UV exposure. Cartogr. Geograph. Information Sci. 2006;33(3):217–31. doi:10.1559/152304006779077318

3. Sliney DH, International Commission on Illumination (CIE). Radiometric quantities and units used in photobiology and photochemistry: recommendations of the Commission Internationale de L'Eclairage (International Commission on Illumination). Photochem. Photobiol. 2007;83(2):425–32. doi:10.1562/2006-11-14-RA-1081

4. Little MP, Tatalovich Z, Linet MS, Fang M, Kendall GM, Kimlin MG. Improving assessment of lifetime solar ultraviolet radiation exposure in epidemiologic studies: comparison of ultraviolet exposure assessment methods in a nationwide U.S. occupational cohort. Photochem. Photobiol. 2018;94(6):1297–307. doi:10.1111/php.12964

5. Wikipedia. Sunlight https://en.wikipedia.org/wiki/Sunlight. Wikipedia. 2018. https://en.wikipedia.org/wiki/Sunlight. Accessed 3/2018.

6. Coste A, Goujon S, Boniol M, et al. Residential exposure to solar ultraviolet radiation and incidence of childhood hematological malignancies in France. Cancer Causes Control. 2015;26(9):1339–49. doi:10.1007/s10552-015-0629-x

7. Chow EJ, Puumala SE, Mueller BA, et al. Childhood cancer in relation to parental race and ethnicity: a 5-state pooled analysis. Cancer. 2010;116(12):3045–53. doi:10.1002/cncr.25099

8. McCullagh P, Nelder JA. Generalized linear models. 2nd edition. Boca Raton, FL: Chapman and Hall/CRC; 1989.

9. R Project version 4.4.2. R: A language and environment for statistical computing. https://www.r-project.org. Vienna, Austria: R Foundation for Statistical Computing; 2024.

10. County Health Rankings. County Health Rankings and Roadmaps (https://www.countyhealthrankings.org/). 2017. Accessed 11/2017.

11. Akaike H. Information theory and an extension of the maximum likelihood principle. In: Petrov BN, Czáki F, editors. 2nd International Symposium on Information Theory. Budapest: Akadémiai Kiadó; 1973. p. 267–81.

12. Akaike H. Likelihood of a model and information criteria. J. Econometrics. 1981;16(1):3–14.

13. Price M, Ballard C, Benedetti J, et al. CBTRUS Statistical Report: Primary Brain and Other Central Nervous System Tumors Diagnosed in the United States in 2017-2021. Neuro Oncol. 2024;26(Supplement_6):vi1–vi85. doi:10.1093/neuonc/noae145

14. Fritz A, Percy C, Jack A, et al. International Classification of Diseases for Oncology, 3rd edition (ICD-O-3). Geneva, Switzerland: World Health Organization; 2000.
