# Supplementary material for: Solar ultraviolet radiation exposure, and incidence of childhood (0–19 years) malignant and non-malignant brain tumour in a US population-based dataset, 2000–2021
Source: Eur J Epidemiol. 2025 Nov 24;41(3):351–66. doi: 10.1007/s10654-025-01314-w (PMC13222178; doi:10.1007/s10654-025-01314-w)
Supplement: Supplementary file 2 — Supplementary file2 (ZIP 67484 KB) [file 10654_2025_1314_MOESM2_ESM.zip › 10654_2025_1314_MOESM2_ESM/Data list.docx]

**Supplement B. Data**

List of data files

| File name | Longer file name |
| --- | --- |
| 1.csv | CNSnonmalignant by subtype, race, sex, age lt 20, 2 yr age_20241229 |
| 2.csv | CNSmalignant by subtype, race, sex, age lt 20, 2 yr age_20241229 |
| 3.csv | uv-county (Tatalovich et al)(master) |
