# Supplementary material for: Solar ultraviolet radiation exposure, and incidence of childhood (0–19 years) malignant and non-malignant brain tumour in a US population-based dataset, 2000–2021
Source: Eur J Epidemiol. 2025 Nov 24;41(3):351–66. doi: 10.1007/s10654-025-01314-w (PMC13222178; doi:10.1007/s10654-025-01314-w)
Supplement: Supplementary file 2 — Supplementary file2 (ZIP 67484 KB) [file 10654_2025_1314_MOESM2_ESM.zip › 10654_2025_1314_MOESM2_ESM/List of R scripts.docx]

**Supplement B. R code.**

**B.1 List of R script files**

| File name | Description |
| --- | --- |
| 1.R | Childhood benign brain and UVR SEER analyses California vs not mk 2A |
| 2.R | Childhood benign brain and UVR SEER analyses interact with age ex sociodemographic mk 2 |
| 3.R | Childhood benign brain and UVR SEER analyses interact with age mk 2 |
| 4.R | Childhood benign brain and UVR SEER analyses leave states out mk 2 |
| 5.R | Childhood Benign Brain and UVR SEER dose resp full adj by major subtypes mk 2 |
| 6.R | Childhood Benign Brain and UVR SEER dose resp mini adj by major subtypes mk 2 |
| 7.R | Childhood Benign Brain and UVR SEER dose response mk 2 |
| 8.R | Childhood Benign Brain and UVR SEER major subtypes full adj analysis mk 2 |
| 9.R | Childhood malignant brain and UVR SEER analyses California vs not mk 2A |
| 10.R | Childhood malignant brain and UVR SEER analyses interact with age ex sociodemographic mk 2 |
| 11.R | Childhood malignant brain and UVR SEER analyses interact with age mk 2 |
| 12.R | Childhood malignant brain and UVR SEER analyses leave states out mk 2 |
| 13.R | Childhood Malignant Brain and UVR SEER dose resp full adj by major subtypes mk 2 |
| 14.R | Childhood Malignant Brain and UVR SEER dose resp mini adj by major subtypes mk 2 |
| 15.R | Childhood Malignant Brain and UVR SEER dose response mk 2 |
| 16.R | Childhood Malignant Brain and UVR SEER major subtypes full adj analysis mk 2 |
| 17.R | Childhood SEER Benign Brain analyses step AIC mk 2 |
| 18.R | Childhood SEER Benign Brain analyses using full set of variables (including UV correlates) mk 2 |
| 19.R | Childhood SEER Benign Brain and UVR analyses by subtype mk 2 |
| 20.R | Childhood SEER Benign Brain and UVR analyses exc 2017-2021 mk 2 |
| 21.R | Childhood SEER Benign Brain and UVR analyses exc sociodemographic mk 2 |
| 22.R | Childhood SEER Benign Brain and UVR analyses excluding Linet specified subtypes mk 2 |
| 23.R | Childhood SEER Benign Brain and UVR analyses mk 2 |
| 24.R | Childhood SEER benign brain and UVR SEER analyses interact median rent mk 2 |
| 25.R | Childhood SEER benign brain and UVR SEER analyses interact racial ethnic mk 2 |
| 26.R | Childhood SEER benign brain and UVR SEER analyses interact sex mk 2 |
| 27.R | Childhood SEER Benign Brain miscellaneous incidence rates by covariates analyses mk 2 |
| 28.R | Childhood SEER Benign Brain miscellaneous incidence rates by subtype race+sex+age mk 2 |
| 29.R | Childhood SEER Malignant Brain analyses step AIC mk 2 |
| 30.R | Childhood SEER Malignant Brain analyses using full set of variables (including UV correlates) mk 2 |
| 31.R | Childhood SEER Malignant Brain and UVR analyses by subtype mk 2 |
| 32.R | Childhood SEER Malignant Brain and UVR analyses exc 2017-2021 mk 2 |
| 33.R | Childhood SEER Malignant Brain and UVR analyses exc sociodemographic mk 2 |
| 34.R | Childhood SEER Malignant Brain and UVR analyses excluding Linet specified subtypes mk 2 |
| 35.R | Childhood SEER Malignant Brain and UVR analyses mk 2 |
| 36.R | Childhood SEER malignant brain and UVR SEER analyses interact median rent mk 2 |
| 37.R | Childhood SEER malignant brain and UVR SEER analyses interact sex mk 2 |
| 38.R | Childhood SEER malignant brain and UVR SEER22 analyses interact racial ethnic mk 2 |
| 39.R | Childhood SEER Malignant Brain miscellaneous incidence rates by covariates analyses mk 2 |
| 40.R | Childhood SEER Malignant Brain miscellaneous incidence rates by subtype race+sex+age mk 2 |
